# Weakening the Interchain Interactions in One Dimensional Cobalt(II) Coordination Polymers by Preventing Intermolecular Hydrogen Bonding

**DOI:** 10.1021/acs.inorgchem.3c01324

**Published:** 2023-06-15

**Authors:** Michał Rams, Thomas Lohmiller, Michael Böhme, Aleksej Jochim, Magdalena Foltyn, Alexander Schnegg, Winfried Plass, Christian Näther

**Affiliations:** † M. Smoluchowski Institute of Physics, Jagiellonian University, Łojasiewicza 11, 30-348 Kraków, Poland; ‡ EPR4Energy Joint Lab, Department Spins in Energy Conversion and Quantum Information Science, Helmholtz-Zentrum Berlin für Materialien und Energie GmbH, Albert-Einstein-Str. 16, 12489 Berlin, Germany; § Institute of Inorganic and Analytical Chemistry, Friedrich Schiller University Jena, Humboldtstraße 8, 07743 Jena, Germany; ∥ Institute of Inorganic Chemistry, Kiel University, Max-Eyth-Straße 2, 24118 Kiel, Germany; ⊥ EPR Research Group, Max Planck Institute for Chemical Energy Conversion, Stiftstraße 34-36, 45470 Mülheim Ruhr, Germany

## Abstract

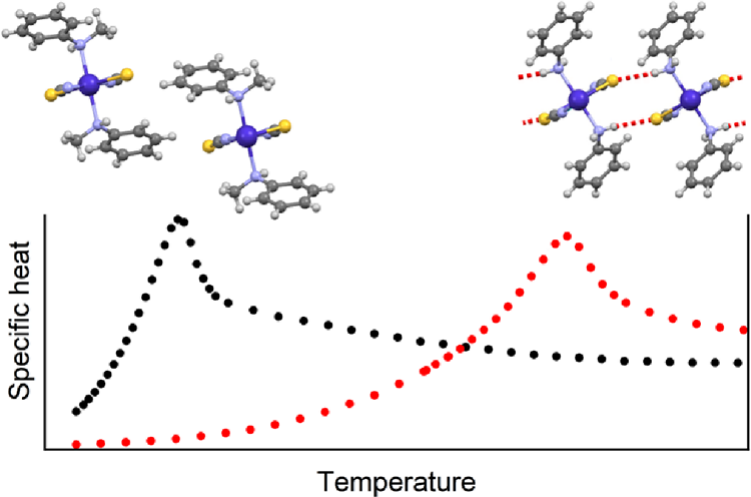

The reaction of Co(NCS)_2_ with *N*-methylaniline leads to the formation of [Co(NCS)_2_(*N*-methylaniline)_2_]_*n*_ (**1**), in which the cobalt(II) cations are octahedrally coordinated and linked into linear chains by pairs of thiocyanate anions. In contrast to [Co(NCS)_2_(aniline)_2_]_*n*_ (**2**) reported recently, in which the Co(NCS)_2_ chains are linked by strong interchain N–H···S hydrogen bonding, such interactions are absent in **1**. Computational studies reveal that the cobalt(II) ions in compound **1** show an easy-axis anisotropy that is lower than in **2**, but with the direction of the easy axis being similar in both compounds. The high magnetic anisotropy is also confirmed by magnetic and FD-FT THz-EPR spectroscopy, which yield a consistent *g_z_* value. These investigations prove that the intrachain interactions in **1** are slightly higher than in **2**. Magnetic measurements reveal that the critical temperature for magnetic ordering in **1** is significantly lower than in **2**, which indicates that the elimination of the hydrogen bonds leads to a weakening of the interchain interactions. This is finally proven by FD-FT THz-EPR experiments, which show that the interchain interaction energy in the *N*-methylaniline compound **1** is nine-fold smaller than in the aniline compound **2**.

## Abstract 2

Replacement of the ligand in [Co(NCS)_2_L_2_]_n_ from L = aniline to L = *N*-methylaniline eliminates intermolecular hydrogen bonding and improves the magnetic behavior.

## Introduction

One-dimensional coordination polymers have been the subject of extensive investigations for many years because of their interesting structural features and their versatile physical properties.^1−4^ In this context, magnetic 1D compounds are of special interest and numerous of those reported in the literature show a variety of magnetic properties like, e.g., spin-crossover,^5−7^ antiferromagnetic (AF) and metamagnetic behavior,^8−11^ ferro- and ferrimagnetism,^12−17^ photomagnetism,^18^ and also single-chain magnet (SCM) behavior.^19−26^ The magnetic properties of most of these compounds are mainly governed by the nature and extent of the intra- and interchain interactions. If the intrachain interactions are ferromagnetic (FM) and strong compared to the interchain interactions and metal ions with large magnetic anisotropy are used, single-chain magnetism may be observed.^27−33^ If the interchain interactions become too strong, three-dimensional magnetic ordering can occur, which is detrimental to SCM behavior. On the other side, this means that in those cases, where the interchain interactions can somehow be controlled, desired magnetic materials may be prepared.

In this context, Aono *et al.* reported on the synthesis of two manganese(III) Schiff-base compounds in which the magnetic chains were connected by ligands that either mediate magnetic exchange or not. As a result, one of them shows SCM behavior, while the second one exhibits AF ordering.^34^ An alternative way to separate magnetic chains more effectively consists in the synthesis of ligands with bulky substituents, which was used, e.g., for the synthesis of an SCM based on a manganese(III) porphyrin tetracyanoethenide.^35^ This strategy was also used for the design of an AF phase of SCMs based on manganese(III) salen-type compounds.^36^ It should be noted that the role of interchain interactions for magnetic ordering has already been discussed by Ostrovsky *et al.,*(37) and that magnetic exchange can also be mediated via hydrogen bonds.^38−41^

In our ongoing work on the synthesis of new magnetic compounds, we have reported on a number of chain compounds with the general composition [Co(NCS)_2_(L)_2_]_*n*_ (L = neutral N-donor coligand), in which the cobalt(II) cations are octahedrally coordinated by two N- and two S-bonding thiocyanate anions and two coligands in the apical position. The cobalt(II) cations are linked by pairs of μ-1,3-bridging anionic ligands into chains, which depending on the metal coordination are linear (all-*trans*) or corrugated (*cis*-*cis*-*trans*).^42^ Independent of the coligand, FM ordering is observed in the linear chains, whereas it is completely suppressed in the corrugated chains.^42^ For the linear chains, two classes of compounds with respect to the magnetic interchain interactions are observed. One of them represents ferromagnets,^43−45^ whereas the others are antiferromagnets that show metamagnetic transitions.^46−50^ In compounds with pyridine derivatives as coligands, the latter show a slow relaxation of magnetization in the AF phase that can be traced back to SCM behavior.^46−51^

To study the influence of the apical coligand on the magnetic anisotropy and the magnetic behavior of these compounds, we also used ligands that are completely different from the pyridine derivatives. With aniline, morpholine, or ethylenethiourea, we obtained compounds with the desired chain topology that at first glance show a magnetic behavior that is very similar to that of the compounds based on pyridine derivatives because for all three compounds, AF ordering and a metamagnetic transition is observed.^52^ Moreover, high-level *ab initio* calculations predict an easy-axis anisotropy for the compound with aniline ([Co(NCS)_2_(aniline)_2_]_*n*_, **2**), similar to what was observed for the compounds with pyridine derivatives. Despite this, surprisingly, no single-chain relaxations are observed in ac susceptibility experiments, which might be attributed to stronger interchain interactions. Initial evidence for this assumption came from the magnetic measurements, where significantly higher-order temperatures were observed compared to the compounds with pyridine derivatives. THz-EPR investigations finally proved that the AF interchain exchange interactions are much stronger than those observed for the pyridine-based chain compounds.^52^ Unlike the pyridine compounds used in our previous studies, aniline is a strong hydrogen bond donor; therefore, the chains are linked by strong intermolecular N–H···S hydrogen bonds, which may be responsible for the deviant magnetic behavior. For the present work, we thus prepared a similar compound based on *N*-methylaniline with one of the two N–H hydrogen atoms replaced by a methyl group, which should lead to a reduction in intermolecular hydrogen bonding and in turn a lowering of the magnetic ordering temperature. Even though the relatively bulky methyl group might hinder coordination to the cobalt(II) cations, we successfully obtained a compound that consists of the desired [Co(NCS)_2_(*N*-methylaniline)_2_]_*n*_ chains (**1**). It was structurally characterized and investigated for its magnetic properties using magnetic and specific heat measurements, computational studies, and THz-EPR spectroscopy.

## Experimental Section

### General

Co(NCS)_2_ was obtained from Sigma Aldrich, while *N*-methylaniline was obtained from Alfa Aesar. All procedures were carried out under ambient conditions if not noted otherwise.

### Synthesis of [Co(NCS)_2_(*N*-Methylaniline)_2_]_*n*_ (**1**)

Powder samples of the compound were prepared by stirring a mixture of Co(NCS)_2_ (1.0 mmol, 175.2 mg) and *N*-methylaniline (4.0 mmol, 432.8 μL) in 0.3 mL of *n*-butanol for 1 day. The residue was filtered, washed with *n*-heptane, and dried in air. Single crystals, which were suitable for X-ray diffraction (SC-XRD), were obtained by reacting Co(NCS)_2_ (0.5 mmol, 87.6 mg) with *N*-methylaniline (1.0 mmol, 108.2 μL) in 0.5 mL of *n*-butanol. Elemental analysis: calc. (%) for C_16_H_18_CoN_4_S_2_ (389.41 g/mol): C, 49.35; H, 4.66; N, 14.39; S, 16.47; found: C, 49.23; H, 4.68; N, 14.08; S, 17.02.

### Single-Crystal Structure Analysis

Data collections were performed using an Imaging Plate Diffraction System (IPDS-2 from STOE) using Mo-Kα radiation. The structure was solved with SHELXT,^53^ and refinement was performed against *F*^2^ using SHELXL-2018.^54^ For all compounds, a numerical absorption correction was performed using X-Red and X-Shape of the software package X-Area.^55^ All non-hydrogen atoms were refined with anisotropic displacement parameters. The C–H hydrogen atoms were positioned with idealized geometry (methyl H atoms were allowed to rotate but not to tip) and were refined isotropically with *U*_iso_(H) = 1.2*U*_eq_(C) (1.5 for methyl H atoms) using a riding model. The N–H hydrogen atom was located in the difference map, its bond length was set to the ideal value, and finally it was refined using a riding model (*U*_iso_(H) = 1.2*U*_eq_(N)). Selected crystal data and details of the structure refinements are given in Table S1.

### Powder X-ray Diffraction

The measurements were performed with Cu Kα_1_ radiation (λ = 1.540598 Å) using a STOE Transmission Powder Diffraction System (STADI P) that is equipped with a MYTHEN 1K detector and a Johansson-type Ge(111) monochromator.

### IR Spectroscopy

The IR data were obtained using an ATI Mattson Genesis Series FTIR Spectrometer, control software: WINFIRST, from ATI Mattson in ATR mode.

### Elemental Analysis

CHNS analysis was performed using a EURO EA elemental analyzer, fabricated by EURO VECTOR Instruments.

### Magnetic Measurements

The measurements were performed using a Quantum Design MPMS 5XL squid magnetometer. Powder samples were frozen in mineral oil. The diamagnetic correction was subtracted.

### Specific Heat Measurements

The measurements were performed by the relaxation technique using a Quantum Design PPMS. The powder samples were pressed into pellets and fixed to a microcalorimeter using Apiezon N grease. The heat capacity of the grease was subtracted.

### Computational Details

A mononuclear structural model [CoZn_2_(NCS)_4_(*N*-methylaniline)_2_]^2+^ (denoted as **Co1**; for depiction see Figure S7) based on the single-crystal structure of **1** has been employed for the *ab initio* computational studies of **1** in which two terminal zinc(II) ions compensate for the negative molecular charge. In addition, the positions of the hydrogen atoms have been optimized at the RI-DFT^56−59^/BP86^60,61^/def2-SVP^62^ level of theory with the Turbomole 7.2 package of programs.^63^ Within this optimization, the paramagnetic cobalt(II) ion was replaced by a diamagnetic zinc(II) ion to achieve a faster SCF convergence and thus to decrease the computational effort. Multireference single-ion CASSCF/CASPT2/RASSI-SO *ab initio* calculations have been performed on the basis of the structural model **Co1** with the OpenMolcas suite of programs in version 18.09.^64^ A scalar-relativistic second-order Douglas–Kroll–Hess Hamiltonian combined with ANO-RCC basis sets (ANO-RCC-VTZP for cobalt and donor atoms; ANO-RCC-VDZ for all remaining atoms) has been utilized to adequately treat relativistic effects.^65−67^ The CASSCF calculations of **Co1** have been performed for all 10 quartet (^4^F, ^4^P) and 40 doublet states (^2^G, ^2^P, ^2^H, ^2^D, ^2^D, ^2^F). The active space consists of 7 electrons in 10 orbitals (3d and 4d shell) to qualitatively improve the CASPT2 energies due to the “double d-shell effect”.^68^ To adequately include the effect of dynamic electron–electron correlation, subsequent CASPT2 calculations based on the CASSCF wave functions were carried out for the 10 quartet and the 12 lowest doublet states (for relative energies see Table S5). Finally, on the basis of the previous CASSCF/CASPT2 wave functions, spin–orbit coupled states were obtained by taking spin–orbit coupling into account via the RASSI-SO approach and allowing a mixing of different spin multiplicities (see Table S6). Magnetic properties such as *g* factors and the orientation of the magnetic axes for the first two Kramers doublets were obtained with the SINGLE_ANISO program (see Table S7). Additionally, the obtained single-ion CASSCF/CASPT2/RASSI-SO multireference data of **Co1** were used employing the POLY_ANISO program^69,70^ to generate more detailed insight into the magnetic structure of the magnetic coordination polymer **1** by utilizing a recently described approach (see Figure 8 and Figures S17–S19).^33^ Corresponding *ab initio* single-ion results of [CoZn_2_(NCS)_4_(aniline)_2_]^2+^ (denoted as **Co2**), which represents the analogous compound **2**, have been taken from the literature.^52^ For **1**, the magnetic exchange constant *J*_Lines_ was determined as described in the literature on the basis of the **Co1** single-ion molecular fragments.^33^ Simulations of the magnetic susceptibility for different *n*-membered spin rings allowed extrapolating the magnetic susceptibility for an infinite chain (*n* → ∞) in the temperature range of 4–50 K for **1** (Figure S18).^33^ In order to obtain the intermolecular interaction in **1**, the Cartesian components of the extrapolated magnetic susceptibility χ_*i*_ (*i* = (*x*, *y*, *z*); χ = (χ_*x*_ + χ_*y*_ + χ_*z*_)/3) were fitted by a mean-field approach (*zJ*′ represents the interchain interaction; *g_i_* values are the *ab initio* Cartesian components of the single-ion *g* factor in the ground state KD):
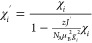
1

### FD-FT THz-EPR Spectroscopy

FD-FT THz-EPR data were acquired at the THz-EPR experiment of the electron-storage ring BESSY II. The setup is described in detail in the literature.^71,72^ Low-α mode linearly polarized coherent synchrotron radiation (CSR)^73^ was used as a broad-band (≈3–50 cm^–1^) THz excitation source. The radiation was transmitted via an evacuated transmission line through a FTIR spectrometer (Bruker IFS 125) and focused on the sample contained in the variable-temperature insert of a 10 T superconducting magnet (Oxford Spectromag). Spectra were recorded in Voigt geometry, with the magnetic field component *B*_1_ of the THz wave perpendicular to the static magnetic field *B*_0_. The transmitted signal was detected by a Si bolometer detector (IR labs) and Fourier-transformed to yield frequency-domain EPR spectra. The sample was prepared by homogenizing in a mortar 56 mg of polycrystalline **1** with 39 mg of polyethylene (PE) powder and pressing it into a pellet. To remove the incident background transmission from the data, referencing was done by dividing raw spectra recorded at different fields by each other, yielding relative transmittance MDS.^72,74^ A first-order polynomial baseline correction was applied to the experimental MDS.

## Results and Discussion

### Synthesis and Crystal Structure of [Co(NCS)_2_(*N*-Methylaniline)_2_]_*n*_ (**1**)

**Figure 1:**
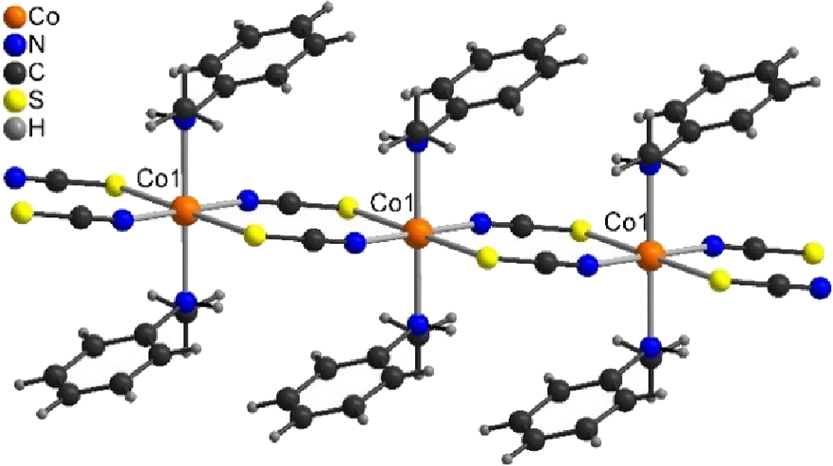
View of a part of a chain in compound **1**.

The synthesis of compound **1** can be achieved by the reaction of cobalt(II) thiocyanate with *N*-methylaniline in small amounts of *n*-butanol or ethanol, with *n*-butanol being preferred as higher yields are obtained in this case. By the reaction in *n*-butanol, we obtained also single crystals, which were characterized by single crystal X-ray diffraction. Compound **1** crystallizes in the monoclinic crystal system with space group *C*2/*c* and *Z* = 4 (Table S1). The asymmetric unit consists of one cobalt(II) cation, which is located on a center of inversion as well as one thiocyanate anion and one *N*-methylaniline coligand in general positions (Figure S1). The cobalt(II) cations are octahedrally coordinated by two N- and two S-bonding thiocyanate anions as well as two coligands in the apical positions. The Co–N_NCS_ and Co–S_NCS_ bond lengths of 2.0438(18) and 2.5790(5) Å, respectively, are slightly shorter than those in the very similar aniline compound **2** (Co–N_NCS_: 2.0554(14) Å and Co–S_NCS_: 2.6089(4) Å), whereas the apical Co–N bond length of 2.2777(19) Å is significantly longer than in the aniline compound (2.1420(15) Å), presumably because of steric reasons (Table S2).^52^Figure S2 shows a structural overlay of the octahedrally coordinated cobalt(II) center of the two compounds. From the bond angles, it is obvious that the coordination octahedra are slightly distorted (Table S2). For compound **1**, the values for the octahedral angle variance and the mean octahedral quadratic elongation amount to σ_θ<oct>_^2^ = 8.0 and λ_oct_ = 1.021, respectively,^75^ which shows that the distortion of the coordination octahedra in **1** is slightly smaller than in **2**, with σ_θ<oct>_^2^ = 13.0 and λ_oct_ = 1.027. The cobalt(II) cations are linked by pairs of μ-1,3-bridging thiocyanate anions into linear chains, which are very similar to those in the analogous compound **2** with aniline as coligand (Figure 1 and Figure S3). The intrachain Co···Co distance of 5.6945 Å for **1** is only slightly longer in comparison with 5.6539 Å for **2**, whereas the smallest interchain Co···Co distance of 8.876 Å for **1** is much longer in comparison with 6.9555 Å for **2** (Table S3).

**Figure 2:**
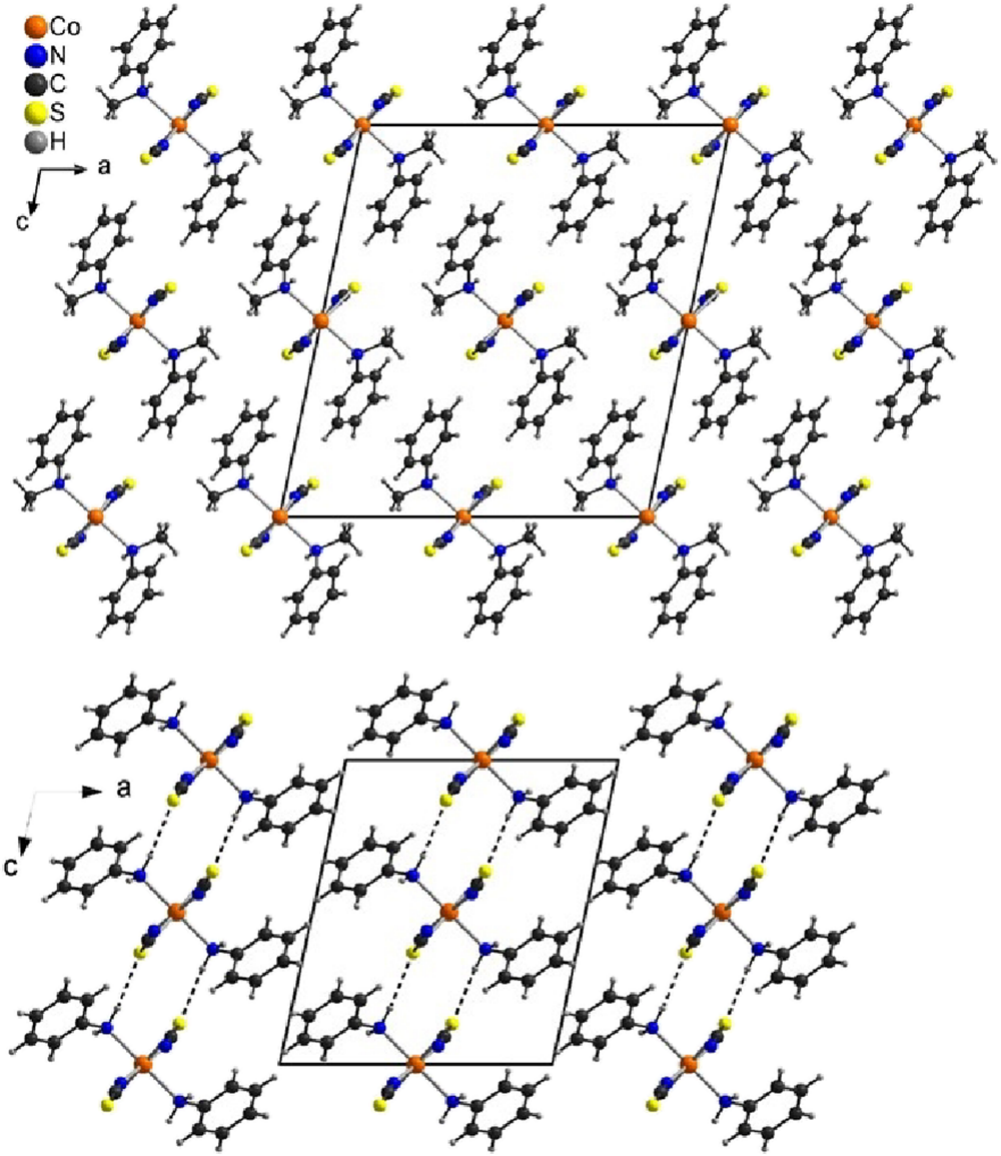
Crystal structure of [Co(NCS)_2_(*N*-methylaniline)_2_]_*n*_ (**1**, top) and of [Co(NCS)_2_(aniline)_2_]_*n*_ (**2**, bottom). Intermolecular N–H···S hydrogen bonding is shown as dashed lines.

A major goal of this work is to test whether the strength of the interchain interactions can be reduced significantly by eliminating the two strong intermolecular N–H···S hydrogen bonds between neighboring chains that are present in the aniline analog (Figure 2, bottom).^52^

In compound **1**, one of the amino hydrogen atoms is replaced by a methyl group that additionally might spatially shield the second N–H hydrogen atom. Surprisingly, the crystal structure of compound **1** is very similar to that of aniline compound **2** (Figure 2). All N_aniline_–Co–N_aniline_ vectors are parallel, and neighboring chains show a very similar arrangement. In contrast to compound **2**, no intermolecular hydrogen bonding is observed in compound **1** and the remaining amino hydrogen atom is involved only in an intrachain N–H···S hydrogen bond to the thiocyanate S atom that coordinates to the same cobalt ion (Figure 2, Figure S3, and Table S4). There are additional C–H···S and C–H···N hydrogen bonds, but they also act within the same chain (Figure S4). All this clearly shows that in compound **1**, all strong interchain interactions are absent and only through-space interactions (dipole–dipole) are possible between chains.

Based on the crystal structure data, a powder pattern was calculated and compared with the experimental pattern, which shows that compound **1** was obtained as a pure phase (Figure S5). It should be noted that in the IR spectrum, the CN stretching vibration is observed at 2109 cm^–1^, consistent with the presence of μ-1,3-bridging anionic ligands (Figure S6).

### Single-Ion Computational Studies

Theoretical investigations have been performed to study the single-ion magnetic properties of the octahedrally coordinated cobalt(II) ion in compound **1** (see Computational Details for more details). The employed *ab initio* CASSCF/CASPT2/RASSI-SO calculations are based on a mononuclear structural model [CoZn_2_(NCS)_4_(*N*-methylaniline)_2_]^2+^ (denoted as **Co1**; for depiction see Figure S7) and reveal a high-spin ^4^T_1g_[^4^F] ground state as expected for cobalt(II) ions in a pseudo-octahedral coordination (see Table S5 for relative CASSCF and CASPT2 energies). By the additional inclusion of spin–orbit coupling, the ^4^T_1g_[^4^F] ground multiplet splits into three so-called Kramers doublets (KDs), and the energetic splitting range is significantly increased from 795 (relative CASPT2 energy) to 1412 cm^–1^ (relative RASSI-SO energy). The first excited spin–orbit coupled state (denoted as KD2) can be found at 230 cm^–1^ (Table S6), which is very similar in comparison to the corresponding value of 228 cm^–1^ for the aniline-based compound **2** as represented by the computational model [CoZn_2_(NCS)_4_(aniline)_2_]^2+^ (denoted as **Co2**).^52^ Consequently, for both compounds, it is expected that at low temperatures, only the ground state KD is thermally populated. This justifies the use of an effective spin Hamiltonian of *S*_eff_ = ^1^/_2_, which considers only the ground-state KD for a single cobalt(II) ion, to describe the magnetic properties of **1**.

**Figure 3:**
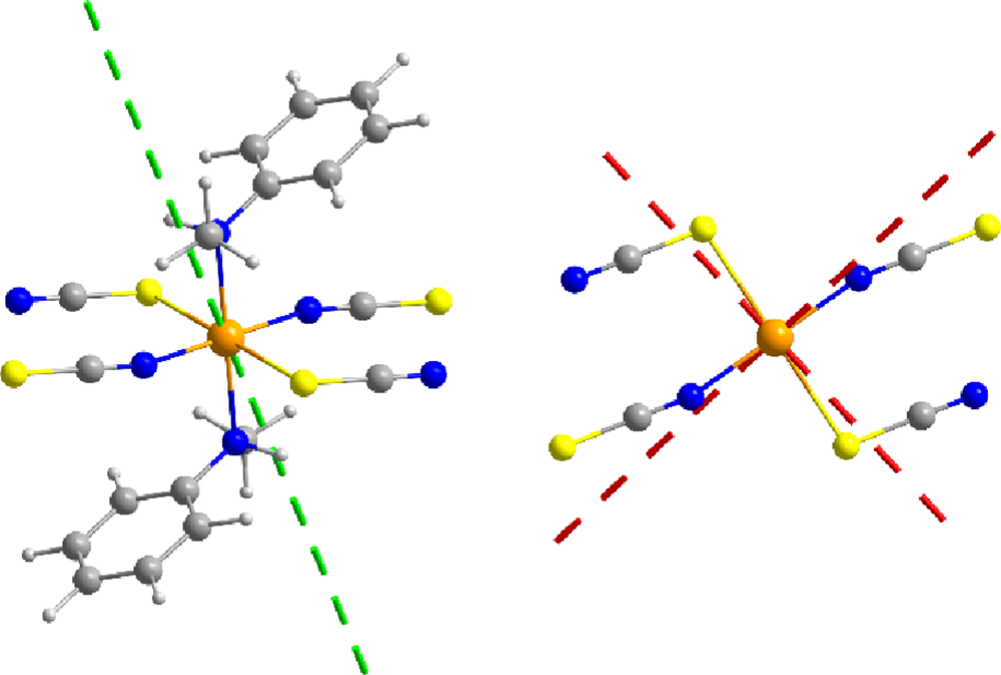
Representation of the magnetic axes of the ground-state Kramers doublet (*S*_eff_ = ^1^/_2_) as obtained from the *ab initio* calculations of **Co1** (left: easy axis of magnetization; right: hard axes of magnetization with a view along the apical donors, which have been omitted for clarity).

The ground-state KD in **Co1** shows a magnetic anisotropy with an easy axis of magnetization (*g_z_* > *g*_*x*,*y*_). The corresponding Cartesian components of the *g* factor for the first two KDs are listed in Table S7. Figure 3 depicts the orientation of the magnetic axes in the ground-state KD of **Co1**. Despite the significant difference in the apical Co–N bond length between **Co1** (2.2777(19) Å) and **Co2** (2.1420(15) Å), the orientation of the magnetic axes is similar in both compounds. The easy axis of magnetization lies within the [N_4_] plane (intersecting angle between plane and easy axis in **Co1**/**Co2**: 1.9/1.2°) and shows an angle of 18.8° (23.1°) with the Co–N bond vector of the axial *N*-methylaniline (aniline) donors for **Co1** (**Co2**). For both compounds, the hard plane of magnetization is quite similar to the [N_2_S_2_] donor plane formed by the four thiocyanate ligands (angle between planes in **Co1**/**Co2**: 22.5°/27.8°). The Cartesian *g_z_* value of 6.600 in **Co1** is significantly smaller than the corresponding value in **Co2** (7.540), which can be attributed to the difference in the apical Co–N bond lengths. In addition, **Co1** shows a larger rhombohedral distortion (*g_x_* ≠ *g_y_*) with respect to the Cartesian *g_x_* and *g_y_* values (*g_x_* = 2.303; *g_y_* = 3.848) compared to **Co2** (*g_x_* = 1.986; *g_y_* = 2.899).^52^ This might be a result of the slightly shorter bond lengths in the basal [N_2_S_2_] donor plane in **1** (*vide supra*), compensating for the significantly elongated Co–N bond lengths in the apical position due to steric reasons. As a consequence, the single-ion magnetic anisotropy in terms of the ratio *g_z_*/*g*_*x*,*y*_ is reduced in **Co1** compared to **Co2** by replacing the second N–H hydrogen atom with a methyl group in the two apical coligands.

### Magnetic and Specific Heat Measurements

**Figure 4:**
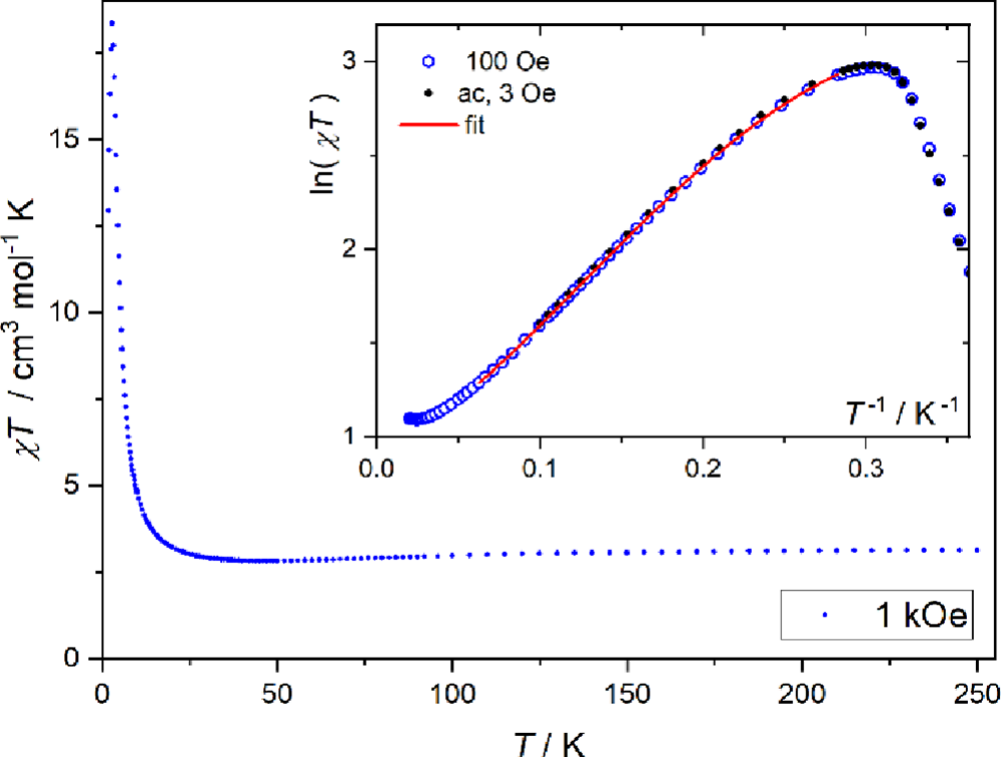
Temperature dependence of magnetic susceptibility for **1** shown as the χ*T* product. Inset: analysis of the low-temperature behavior using the Ising chain model (see text).

The magnetic susceptibility, χ, for **1** is shown as the χ*T* product in Figure 4. The χ*T* value at 250 K is 3.2 cm^3^ mol^–1^ K, similar to other systems of cobalt(II) in an octahedral N_4_S_2_ coordination. With *T* decreasing from room temperature, χ*T* slightly decreases, which is related to the single ion properties of cobalt(II).^76^ Below 40 K, χ*T* starts to increase, which shows that the exchange interaction along the Co(NCS)_2_ chains is FM, similar to all Co(NCS)_2_L_2_ linear chain compounds reported so far.

**Figure 5:**
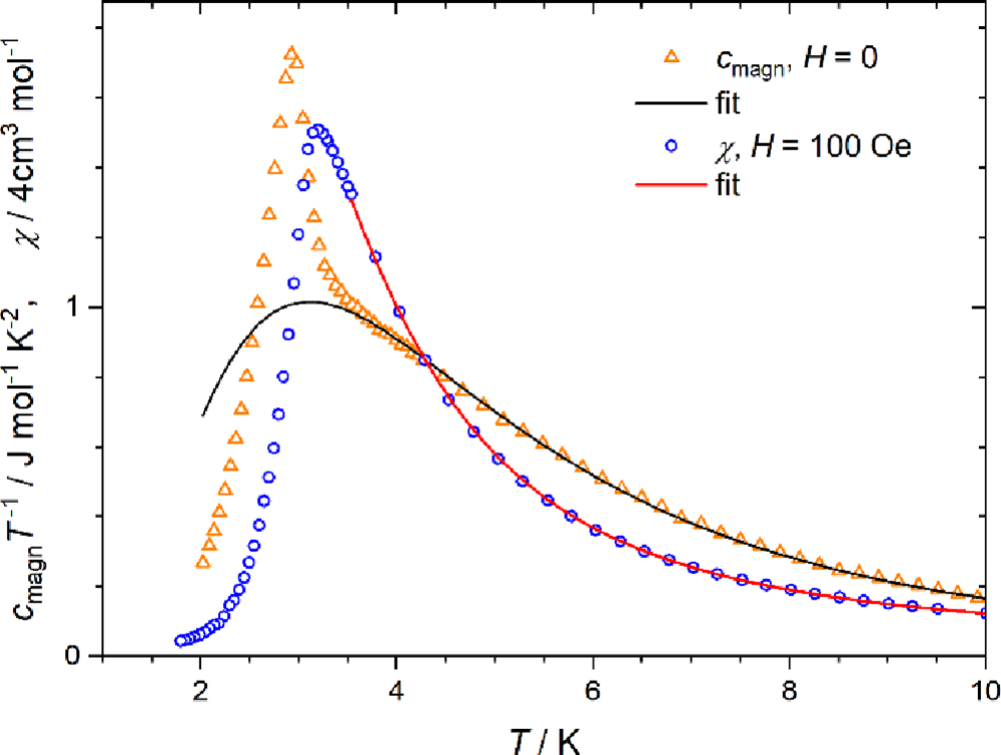
Magnetic contribution to specific heat (triangles) and magnetic susceptibility (circles) for **1**. Both quantities are fitted using the Ising chain model (see text).

However, an AF state is reached at the lowest temperatures, which is demonstrated by the peak visible in the χ(*T*) dependence measured at low field (Figure 5). There is no difference between zero-field cooled and field cooled susceptibility (Figure S8). The critical temperature of the AF ordering in **1** is 3.00(5) K, as determined from the maximum of d(χ*T*)/d*T*.^77^ The AF ground state of FM chains has to be induced by an AF interaction between the chains, and such interaction can be of both origins, dipolar and exchange, such as elucidated for [Co(NCS)_2_(4-methoxypyridine)_2_]_*n*_.^46^

**Figure 6:**
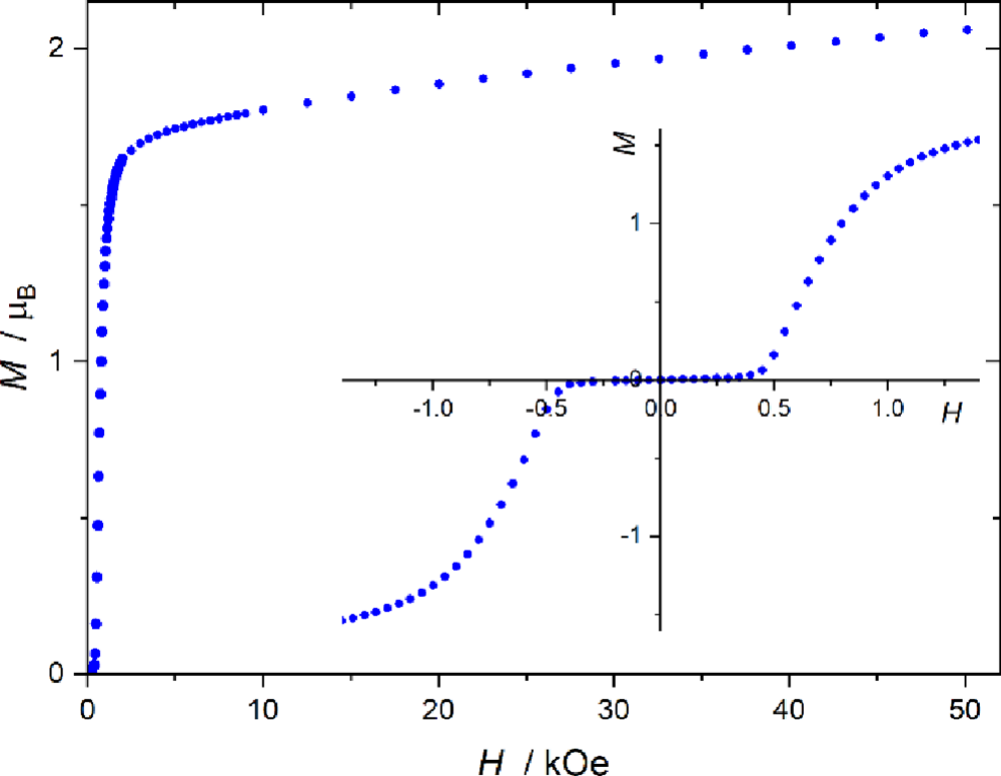
Field dependence of magnetization measured for **1** at 1.8 K. Inset: zoom of the low field range.

The field dependence of the magnetization for **1** shows a metamagnetic transition that further confirms the AF ground state (Figure 6). At *T* = 1.8 K, the critical field of this transition (the d^2^*M*/d*H*^2^ maximum) is at *H_c_* = 430 Oe. The *M*(*H*) dependence is still not saturated at 50 kOe in accordance with the high anisotropy of the ground state of the cobalt(II) ions in **1**, as shown by *ab initio* calculations (*vide supra*).

The specific heat, *c*, measured at zero field in the 2–40 K temperature range is shown in Figure S9, while the magnetic contribution (obtained by subtracting the estimated lattice contribution) is shown in Figure 5. The peak of *c*_magn_(*T*) present at *T_c_* = 2.98(3) K is related to the second-order magnetic ordering transition. This *T_c_* value is in perfect agreement with that determined from susceptibility data (*vide supra*). The *c*_magn_(*T*) bump around 4 K is related to exchange interaction in the cobalt(II) spin chain, while the lattice contribution, *c*_latt_, dominates the specific heat above 10 K.

Quantitative analysis of low *T* susceptibility and specific heat as presented below is done assuming that the effective exchange interaction between cobalt(II) in the ground-state Kramers doublet (*S*_eff_ = 1/2) is of the Ising type, as anticipated by *ab initio* calculations described above. For the Ising spin chain model with the Hamiltonian
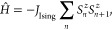
2analytical solutions for the zero-field susceptibility and specific heat are available.^78^ Full equations used for the fitting are the same as given in ref (79). The fit of the susceptibility in the 3.5–20 K range yields *J*_Ising_ = 15.7(2) cm^–1^, the factors *g_z_* = 6.8(1) and *g_x,y_* = 3.1(4), the mean-field interchain interaction *zJ′* = −0.37(1) cm^–1^, and the temperature-independent paramagnetic contribution χ_0_ = 0.011(2) cm^3^ mol^–1^. The last term includes the influence of the next Kramers doublet on the susceptibility in the low *T* limit. The fitted susceptibility dependence is shown in the inset of Figure 4 as ln(χ*T*)(1/*T*) and in Figure 5 as χ(*T*). Similar analysis of the specific heat using the same Hamiltonian requires simultaneous fitting of the phonon contribution, which is here approximated using a linear combination of Debye and Einstein phonon models. The fit in the temperature range 3.5–40 K leads to *J*_Ising_ = 14.0(2) cm^–1^, for which the simulated curve is shown in Figure 5 (see Figure S9 for the full range). Below 3.5 K, this curve deviates from the experimental points because of the onset of the 3-dimensional ordering, which cannot be accounted for by the one-dimensional model.

To check for expected magnetic relaxations, we measured the ac susceptibility for frequencies from 1 to 1000 Hz. The temperature dependence measured at zero dc field is shown in Figure S10. A slight frequency dependence of χ*′* is observed below 2.1 K, but χ″ remains close to zero. Higher ac susceptibility is observed when a dc field is applied to move the AF ordered system in the metamagnetic transition range. Such behavior of a system of AF-ordered FM chains is explained in ref (46) by micromagnetic Monte Carlo simulations. The ac susceptibility measured for **1** at *H_dc_* = 500 Oe and its analysis using a Cole–Cole model are shown in Figure S11. The energy barrier determined assuming the Arrhenius dependence τ = τ_0_ exp(Δ_τ_/*k*_B_*T*) is Δ_τ_ = 25(2) cm^–1^ with the preexponential factor τ_0_ = 0.5(1) ns (Figure S12). The relaxation in **1** at 1.8 K is over 10^3^ times slower than for [Co(NCS)_2_(methoxypyridine)_2_]_*n*_ under similar conditions.

### FD-FT THz-EPR Spectroscopy

As we have shown in previous work on magnetic chains, frequency-domain Fourier-transform (FD-FT) THz-EPR, directly probing magnetic dipole transitions, is excellently suited to determine the energy difference between the ground and first excited spin states as well as corresponding *g* values.^42,46,52^ In particular, we could establish an experimental method to deconvolute contributions from the intrachain and the interchain exchange couplings to the measured energy gaps. Here, we apply the same approach to characterize the interaction energies in **1** and compare the results with those for the corresponding aniline compound **2**.

**Figure 7:**
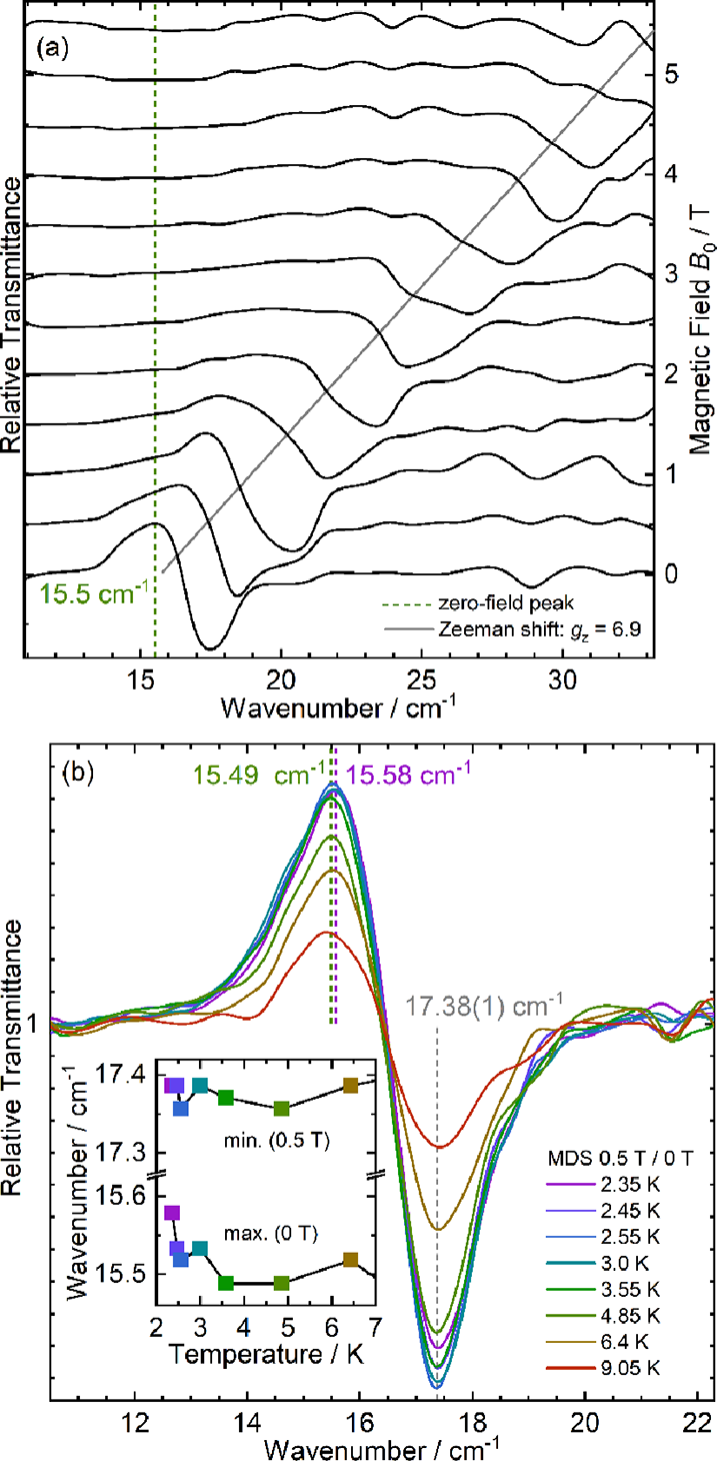
FD-FT THz-EPR spectra of **1**. (a) Field dependence at 4.8 K measured with an experimental resolution of 1 cm^–1^. The MDS (black solid lines) were calculated by division of a raw spectrum at *B*_0_ + 0.5 T by one measured at *B*_0_, such that maxima correspond to stronger absorption at lower *B*_0_ and minima correspond to increased absorption at higher *B*_0_. (b) Temperature dependence from 2.35 to 9.05 K at an experimental resolution of 0.5 cm^–1^, presented as MDS (0.5 T/0 T). Inset: EPR energies for the ground-to-first-excited-state transitions obtained as the positions of the peak maxima, corresponding to *B*_0_ = 0 T (bottom), and peak minima, corresponding to *B*_0_ = 0.5 T (top).

Field-dependent low-temperature (4.8 K) FD-FT THz-EPR spectra of a pressed powder sample of **1** are depicted as magnetic-field division spectra (MDS) up to 6 T/5.5 T in Figure 7a. As evidenced by the position of the peak maximum in the 0.5 T/0 T spectrum, the zero-field transition energy between the ground and the first excited state of chain **1** is Δ_chain_ ≈ 15.5 cm^–1^ (*T* > *T*_c_). A *g_z_* value of 6.9 can be extracted from the most prominent Zeeman shift of the signal to higher energies with increasing field *B*_0_, illustrated by the gray line in Figure 7a. Assuming the Ising spin chain Hamiltonian as above, the energy of the first excited state, i.e., the energy required to create two domain walls, is Δ_chain_ = *J*_Ising_. Δ_chain_ and *g_z_* derived from THz EPR are in excellent agreement with *J*_Ising_ = 15.7(2) cm^–1^ and *g_z_* = 6.8(1) as obtained from the analysis of the low-temperature susceptibility.

The temperature dependence of the ground-to-first-excited-state EPR signals of **1** is presented in Figure 7b in the form of 0.5 T / 0 T MDS, wherein the peaks pointing upward correspond to absorption by magnetic transitions at 0 T, whereas downward peaks correspond to absorption processes at 0.5 T.

The inset displays the resonance energies, obtained as the upward and downward peak positions, plotted against the experimental temperature. The 0.5 T resonance varies slightly around an average energy of 17.38(1) cm^–1^ but does not exhibit a systematic change with temperature. In contrast, the 0 T line shifts from 15.58 to 15.49 cm^–1^ between 2.35 and 3.55 K. Such a difference results from the metamagnetic transition, as we have shown before for several cobalt(II) coordination polymers.^42,46,52^ While in the unordered state above *T*_c_, only the intrachain exchange energy Δ_chain_ ≈ 15.5 cm^–1^ is required to excite the spin, and 3D ordering below *T*_c_ additionally requires the interchain interaction energy Δ_inter_ = 0.09(2) cm^–1^ for the transition to the excited state. The temperature range of the shift is centered right around *T*_c_ = 3.0 K determined from specific heat and susceptibility. Employing the resonance condition and *g_z_* = 6.9, Δ_inter_ corresponds to a critical field *H*_c_(EPR) = Δ_inter_/(*g_z_*μ_B_) = 280(60) Oe. The discrepancy to *H*_c_ = 430 Oe at 1.8 K from magnetometry is probably due to instrumental limitations as 2.35 K represent the minimum reachable sample temperature. The constant resonance energy at 0.5 T is hence a result of effective decoupling of the spin chains at fields higher than *H*_c_.

In summary, the FD-FT THz-EPR experiments reveal very small interchain interaction energies in **1**, amounting to below 1% of the intrachain exchange interaction energy. A similarly small ratio Δ_inter_/Δ_chain_ = 0.006 has for instance been determined for the SCM [Co(NCS)_2_(methoxypyridine)_2_]_*n*_,^46^ while the corresponding aniline compound exhibits a six-fold larger ratio Δ_inter_/Δ_chain_ = 0.036.^52^

### Single-Chain Computational Studies

In a recent study, we demonstrated a theoretical approach to investigate magnetic domains in FM one-dimensional coordination polymers based on *ab initio* multireference calculations of single-ion paramagnetic centers.^33^ This method employs a magnetic exchange coupling scheme of *n*-membered spin rings to approximate the behavior of a spin chain. In the case of a 12-membered spin ring, seven consecutive spin multiplets with an energy separation of *J*_calc_ result for an ideal Ising anisotropy of the spins (see Figure S13). Furthermore, all spin microstates belonging to the same multiplet are degenerate for the ideal Ising case.

For this work, we applied the single-ion *ab initio* models **Co1** and **Co2** to the theoretical approach of *n*-membered spin rings in combination with FM exchange (*J* > 0). It turns out that for both single-ion models **Co1** and **Co2**, representing the coordination polymers **1** and **2**, respectively, a strong deviation from ideal Ising behavior is apparent due to the large shift in energies of the spin microstates (see Figure S13 for a depiction of the spin microstates). The lower single-ion magnetic anisotropy in **Co1** compared to **Co2** leads to a broader distribution of spin microstates for **Co1** in which the individual spin microstates cannot be assigned to the corresponding spin multiplets. This indicates a strong deviation from the ideal Ising behavior for **Co1**. In contrast, for **Co2**, the seven individual spin multiplets can still be recognized by the depiction of the spin states, even though a distinct deviation from ideal Ising behavior is apparent.

As demonstrated in our previous work,^33^ the experimental magnetic susceptibility data at low-temperatures can be utilized to determine the intrachain magnetic coupling constant for the Lines model^80^ on the basis of computational results for the single ion. For **1**, an FM coupling constant of *J*_Lines_ = 2.91 cm^–1^ (*S*_eff_ = 3/2) was obtained by a fit of the simulated curve to the experimental data in the temperate range of 8–50 K with the POLY_ANISO program based on the **Co1** computational results for *n*-membered spin rings (see Figure S14). Furthermore, a *g_z_* value of 6.54 could be obtained for *n* = 12 for the ground-state doublet, which is similar to the single ion *g_z_* value (6.600) obtained for **Co1** and the *g_z_* value of 6.8(1) obtained by a fit of the experimental magnetic susceptibility using the Ising model (vide supra). For **1**, a corresponding Ising coupling constant *J*_calc_ of 16.06 cm^–1^ was determined by taking the energy of the two highest spin microstates, which correspond to an energy of 6*J*_calc_ in the case of a coupling scheme of a 12-membered spin ring. This is consistent with the Ising coupling constant *J*_Ising_ as determined by a fit of the magnetic susceptibility data (15.7(1) cm^–1^) and FD-FT THz-EPR experiments (15.5 cm^–1^) for **1**. The slight overestimation of about 4% for the intramolecular Ising coupling *J*_calc_ might be due to an underestimation of the *g_z_* value obtained from the *ab initio* calculations, which is compensated for by a higher *J*_calc_ value when the magnetic susceptibility is fitted.

**Figure 8:**
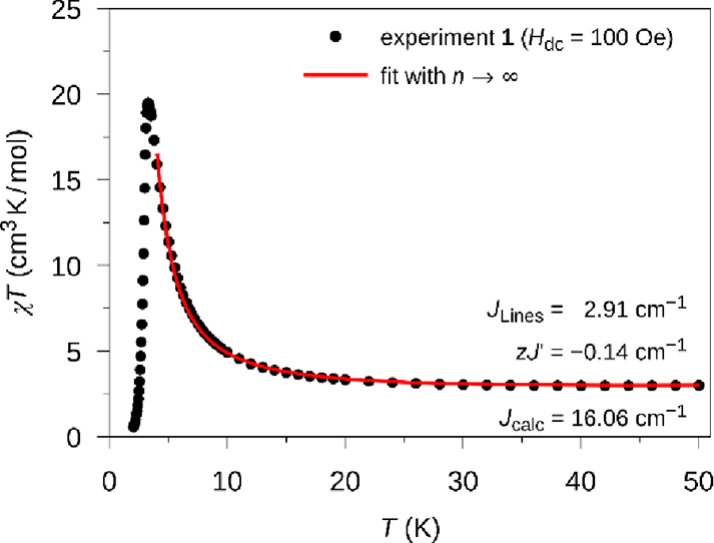
Temperature dependence of the experimental magnetic susceptibility for **1** under an applied dc field of 100 Oe (black circle). The red line represents a fit of an *n*-membered spin ring with *n* → ∞ based on the mononuclear *ab initio* model **Co1** together with an interchain interaction *zJ′* in the temperature range of 4–50 K.

Once *J*_Lines_ is determined, simulations of magnetic susceptibility for different *n*-membered spin rings on the basis of the single-ion *ab initio* results can be performed (Figure S15). The simulations for different values of *n* allow extrapolation of the magnetic susceptibility data for an infinite chain (*n* → ∞).^33^ Below approximately 6 K, the theoretical magnetic susceptibility data for an infinite chain and the experimental data begin to diverge significantly (Figure S15), indicative of the presence of intermolecular interactions such as dipole–dipole interactions. To determine the intermolecular interactions, the theoretical magnetic susceptibility data extrapolated for an infinite chain were used in a further approach in combination with an interchain interaction described by a mean-field approach (*zJ*′) and the Cartesian components of the single-ion *g* factor as obtained by the *ab initio* calculations (see Computational Details). From the temperature range of 4–50 K, an AF interchain interaction of *zJ*′ = −0.14 cm^–1^ was obtained (Figure 8). Thus, the resulting ratio of inter- and intramolecular interactions in **1** of |*J*′/*J*_calc_| = 0.004 (assuming *z* = 2 with *J′* = −0.07 cm^–1^) is in good accordance with the value obtained by the FD-FT THz-EPR experiments (Δ_inter_/Δ_intra_ = 0.006) for **1**. Below 4 K, the extrapolation of the magnetic susceptibility data (*n* → ∞) based on the employed *n*-membered spin ring approach becomes too inaccurate due to model and hardware limitations (*n*_max_ = 12) and hence a temperature of 4 K was used as the lower temperature limit for the fit of *zJ*’.

### Comparison of **1** and **2**

As mentioned above, these investigations originate from previous results obtained for the aniline compound **2**, where strong interchain N–H···S interactions to one of the amino H atoms is observed. Therefore, the aniline ligand was replaced by *N*-methylaniline, where one of the N–H hydrogen atoms is replaced by a methyl group, which leads to similar chains in which the cobalt(II) cations are octahedrally coordinated by two coligands in the apical positions and two N- and two S-bonding thiocyanate anions in the basal plane. In compound **1**, the bond lengths to the apical coligands are longer and those to the thiocyanate anions are shorter than in compound **2** (Table 1), which might originate from some steric repulsion of the bulky methyl group.

**Table 1:** Comparison of Selected Data for the Compounds [Co(NCS)_2_(L)_2_]_*n*_**1** and **2**

	**1** (L = *N*-methylaniline)	**2** (L = aniline)
Co–N_coligand_	2.2777(19) Å	2.1420(15) Å
Co–N_NCS_	2.0438(18) Å	2.0554(14) Å
Co–S_NCS_	2.5790(5) Å	2.6089(4) Å
*g_x_* (calc.)	2.303	1.986
*g_y_* (calc.)	3.848	2.899
*g_z_* (calc.)	6.600	7.540
∠(easy axis/Co–N_coligand_)	18.8°	23.1°
*T_c_* (magn.)	3.00 K	6.44 K
*H_c_* (magn.)	430 Oe	not applicable
*J*_Ising_ (magn.)	15.7 cm^–1^	not determined
Δ_τ_	25 cm^–1^	not observed
Δ_chain_ (EPR)	15.5 cm^–1^	22.5 cm^–1^
Δ_inter_ (EPR)	0.09(2) cm^–1^^a^	0.8 cm^–1^

a The error margin is based on the standard deviation of the resonance energies at 0.5 T (17.38(1) cm^–1^, Figure 7), which do not exhibit a shift induced by the metamagnetic transition.

*Ab initio* CASSCF/CASPT2/RASSI-SO calculations using a structural model for **1** prove an easy-axis anisotropy (*g_z_* > *g*_*x*,*y*_), as was the case for **2**, with a similar orientation of the magnetic easy axis, lying in the [N_4_] plane for both compounds and showing an angle of around 20° with the Co-N_coligand_ vector (Table 1). In summary, the replacement of one N–H hydrogen atom by a methyl group significantly reduces the single-ion magnetic anisotropy in compound **1**. This is consistent with previous investigations, indicating that magnetic anisotropy decreases with increasing donor strength of the apical coligand. As mentioned above, the arrangement of the chains is very similar in **1** and **2**, despite the fact that in the *N*-methylaniline compound, interchain N–H···S hydrogen bonding is absent. Consequently, it can be assumed that this leads to significantly weaker magnetic interchain interactions. This is already evident from the critical temperature of the magnetic ordering (Table 1), which is significantly lower for the *N*-methylaniline compound **1** than for the aniline compound **2**, as determined by specific heat (Figure S16) and low-field susceptibility measurements (Figure S17). For both compounds, the intrachain interaction is FM (Figure S18) and reaches an AF ground state at lower temperatures, showing that the interchain interactions must be AF. In line with this, a metamagnetic transition was observed for **1** at a critical field of *H_c_* = 430 Oe, but this was not possible for **2** because in this case already, a small field induces an FM component and a magnetic hysteresis is observed (Figure S19). Our previous investigations for **2** indicate that the dominant interaction mediated by interchain hydrogen bonding is FM, while a much weaker interaction between FM planes is AF.^52^ The *M*(*H*) dependence for both compounds is not saturated at 50 kOe (Figure S20), which is consistent with the high magnetic anisotropy, as shown by *ab initio* calculations.

Analysis of the low-temperature susceptibility leads to *g_z_* = 6.8(1) and *J*_Ising_ = 15.7(2) cm^–1^ for **1**, which is in perfect agreement with the FD-FT THz-EPR investigations (Table 1). Compound **2** deviates from quasi-one dimensional behavior to such an extent that the methods used in this paper to simulate the susceptibility of **1** fail to fit the susceptibility of **2**. Therefore, the value of the intrachain interactions for **2** could only be determined from THz-EPR data, which lead to a value significantly higher than for **1** (Table 1). Regarding the main question of this work, whether the interchain interactions can be weakened or eliminated by preventing intermolecular hydrogen bonding, our FD-FT-THz-EPR experiments clearly demonstrate that the energy of the interchain exchange interactions is about an order of magnitude lower in the *N*-methylaniline compound **1** compared to the aniline compound **2** (Table 1).

## Conclusions

This work originates from previous studies on a [Co(NCS)_2_(L)_2_]_*n*_ chain compounds based on aniline as the coligand, which in comparison to related pyridine-based compounds shows much stronger interchain interactions, evidently due to strong intermolecular N–H···S hydrogen bonding. In this work, one of the N–H hydrogen atoms was replaced with a methyl group, resulting in a compound with the same chain structure and a similar arrangement of the chains in the crystal while completely lacking intermolecular hydrogen bonding, which strongly affects the magnetic interchain interactions. *Ab initio* calculations for a pseudo-octhahedrally coordinated single cobalt(II) ion in **1** revealed weaker magnetic anisotropy compared to the aniline analogue **2**, which was attributed to the significantly elongated apical Co–N bond lengths of the two coligands. The theoretically determined magnetic parameters for **1** agree well with the magnetic properties obtained by experimental methods. This includes the presence of significant magnetic anisotropy consistent with the *g* factor *g_z_* of the ground state of the cobalt(II) ion obtained from susceptibility analysis and confirmed by THz-EPR spectroscopy. Both experimental techniques provide an almost identical value for the intrachain interaction, and from the spectroscopic measurements, it was found that the intrachain exchange in the *N*-methylaniline compound is lower than in the aniline analog. Initial evidence that the interchain magnetic interactions are significantly reduced for **1** came from the magnetic measurements, where a much lower critical temperature for the magnetic ordering and a metamagnetic transition at low field was observed for the *N*-methylaniline compound. This was further confirmed by THz-EPR measurements, which showed that the interchain interaction is lower in the *N*-methylaniline compound by nearly an order of magnitude. Concerning the strategy that was used to influence the magnetic interchain interactions, it is noted that it is only useful for the modification of compounds, where it is likely that the magnetic properties are influenced or even governed by intermolecular hydrogen bonding. Even if this is sometimes difficult to predict, one can try by using ligands, in which the donor or acceptor groups are replaced by others that can prevent hydrogen bonding. In summary, this work clearly indicates that the magnetic properties can be controlled to some extent by controlling the intermolecular interactions such as hydrogen bonding in this class of chain compounds via the coligand used.

## Supporting Information Available

Details of structure determinations, ORTEP plot, comparison of crystal structures, hydrogen bonds, PXRD and IR spectra, details of *ab initio* calculations, results of specific heat, dc and ac magnetic measurements, and details of spin ring calculations (PDF)
