# Supplementary material for: Weakening the Interchain Interactions in One Dimensional Cobalt(II) Coordination Polymers by Preventing Intermolecular Hydrogen Bonding
Source: Inorg Chem. 2023 Jun 15;62(26):10420–30. doi: 10.1021/acs.inorgchem.3c01324 (PMC10324318; doi:10.1021/acs.inorgchem.3c01324)
Supplement: Supplementary file 1 — ic3c01324_si_001.pdf [file ic3c01324_si_001.pdf]

# Supporting Information

## Weakening the Interchain Interactions in 1D Cobalt(II) Coordination Polymers by Preventing Intermolecular Hydrogen Bonding

Michał Rams[a]*, Thomas Lohmiller[b,c]*, Michael Böhme[d], Aleksej Jochim[e], Magdalena Foltyn[a], Alexander Schnegg[b,f], Winfried Plass[d]*, and Christian Näther[e]*

[a] *M. Smoluchowski Institute of Physics, Jagiellonian University, Łojasiewicza 11, 30-348 Kraków, Poland*

[b] *EPR4Energy Joint Lab, Department Spins in Energy Conversion and Quantum Information Science, Helmholtz-Zentrum Berlin für Materialien und Energie GmbH, Albert-Einstein-Str. 16, 12489 Berlin, Germany*

[c] *Present address: Institut für Chemie, Humboldt-Universität zu Berlin, Brook-Taylor-Straße 2, 12489 Berlin, Germany*

[d] *Institute of Inorganic and Analytical Chemistry, Friedrich Schiller University Jena, Humboldtstraße 8, 07743 Jena, Germany*

[e] *Institute of Inorganic Chemistry, Kiel University, Max-Eyth-Straße 2, 24118 Kiel, Germany*

[f] *EPR Research Group, MPI for Chemical Energy Conversion, Stiftstraße 34-36, 45470 Mülheim Ruhr, Germany*

*Emails:
m.rams@uj.edu.pl
thomas.lohmiller@helmholtz-berlin.de
sekr.plass@uni-jena.de
cnaether@ac.uni-kiel.de

## Content


| | | |
|---|---|---|
| Tab. S1 | Selected crystal data and details of the structure refinements for compound **1**. | S3 |
| Fig. S1 | Crystal structure of **1** with labelling and displacement ellipsoids. | S4 |
| Tab. S2 | Bond lengths and angles for **1**. | S4 |
| Tab. S3 | Shortest intrachain and interchain distances in **1** and in **2**. | S4 |
| Fig. S2 | Structural overlay of octahedral cobalt(II) center in **1** and **2**. | S5 |
| Fig. S3 | View of a part of a chain in **1** and of **2**. | S5 |
| Fig. S4 | View of a part of a chain of **1** with intramolecular C–H···S and C–H···N hydrogen bonding shown. | S6 |
| Tab. S4 | Hydrogen bonds for **1**. | S6 |
| Fig. S5 | Experimental and calculated PXRD pattern of **1**. | S7 |
| Fig. S6 | IR spectrum of **1**. | S7 |
| Fig. S7 | Mononuclear *ab initio* computational model for **1**. | S7 |
| Tab. S5 | Relative CASSCF and CASPT2 energies for the computational model **Co1**. | S8 |
| Tab. S6 | Relative energies for the Kramers doublets of the $^4T_{1g}$ multiplet for **Co1**. | S9 |
| Tab. S7 | Cartesian components of the *g* tensor ($S_{eff}$ = ½) for **Co1**. | S9 |
| Fig. S8 | Zero-field cooled and field cooled susceptibility for **1** measured at 30 Oe. | S10 |
| Fig. S9 | Specific heat for **1** in the temperature range of 2-40 K as used for the analysis. | S10 |
| Fig. S10 | Temperature dependence of ac susceptibility for **1** at zero dc field. | S11 |
| Fig. S11 | Ac susceptibility measured for **1** at dc field of 500 Oe and its analysis using single mode Cole–Cole model. | S11 |
| Fig. S12 | Magnetic relaxation times for **1** at $H_{dc}$ = 500 Oe with the fitted Arrhenius equation. | S12 |
| Fig. S13 | Depiction of spin states as obtained by POLY_ANISO simulations for **1** and **2**. | S12 |
| Fig. S14 | Calculated magnetic susceptibility for **1** based on a simulation of a 12-membered spin ring to determine $J_{Lines}$. | S13 |
| Fig. S15 | Calculated magnetic susceptibility for **1** based on simulations of *n*-membered spin rings without an interchain interaction ($zJ$ = 0). | S13 |
| Fig. S16 | Comparison of specific heat for **1** and **2**. | S14 |
| Fig. S17 | Comparison of susceptibility temperature product for **1** and **2**. | S14 |
| Fig. S18 | Comparison of low field susceptibility for **1** and **2**. | S15 |
| Fig. S19 | Comparison of field dependence of magnetization at 1.8 K for **1** and **2**. | S15 |
| Fig. S20 | Comparison of high-field magnetization at 1.8 K for **1** and **2**. | S16 |

Table S1. Selected crystal data and details of the structure refinements for compound **1**.

| **Compound** | $[Co(NCS)_2(N\text{-methylaniline})_2]_n$ **(1)** |
|---|---|
| Formula | $C_{16}H_{18}CoN_4S_2$ |
| MW / g mol$^{-1}$ | 389.39 |
| Crystal system | *monoclinic* |
| $a$ / Å | 16.8142(9) |
| $b$ / Å | 5.6945(2) |
| $c$ / Å | 18.2861(10) |
| $\alpha$ / ° | 90 |
| $\beta$ / ° | 101.986(4) |
| $\gamma$ / ° | 90 |
| $V$ / Å$^3$ | 1712.69(15) |
| $T$ / K | 200 |
| Space group | $C2/c$ |
| Z | 4 |
| $D_{calc}$ / g cm$^{-3}$ | 1.510 |
| $\mu$ / mm$^{-1}$ | 1.249 |
| Min/max. transm. | 0.8560/0.7660 |
| $\theta_{max}$ / deg | 27.002 |
| measured refl. | 5411 |
| $R_{int}$ | 0.0275 |
| unique refls. | 1856 |
| refl. $[F_0 > 4\sigma(F_0)]$ | 1574 |
| parameters | 108 |
| $R_1$ $[F_0 > 4\sigma(F_0)]$ | 0.0363 |
| $wR_2$ [all data] | 0.1017 |
| GOF | 1.060 |
| $\delta$ / eÅ$^{-3}$ | 0.504/-0.405 |

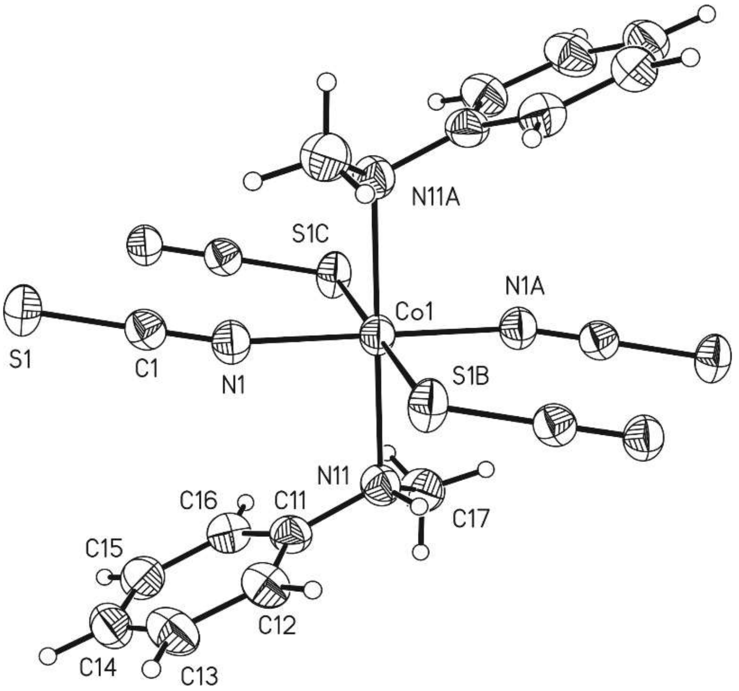


Fig. S1: Crystal structure of **1** with labelling and displacement ellipsoids drawn at the 50% probability level. Symmetry transformations used to generate equivalent atoms: A: −*x*+1, −*y*+1, −*z*+1; B: *x*, *y*+1, *z* and C: −*x*+1, −*y*, −*z*+1.

Table S2. Bond lengths (Å) and angles (°) for **1**.

| | | | |
|---|---|---|---|
| Co1–N1A | 2.0438(18) | Co1–N11A | 2.2777(19) |
| Co1–N1 | 2.0438(18) | Co1–S1B | 2.5790(5) |
| Co1–N11 | 2.2777(19) | Co1–S1C | 2.5790(5) |
| | | | |
| N1A–Co1–N1 | 180.00 | N11–Co1–S1B | 87.81(5) |
| N1A–Co1–N11 | 86.20(7) | N11A–Co1–S1B | 92.19(5) |
| N1–Co1–N11 | 93.80(7) | N1A–Co1–S1C | 88.36(5) |
| N1A–Co1–N11A | 93.80(7) | N1–Co1–S1C | 91.64(5) |
| N1–Co1–N11A | 86.20(7) | N11-Co1–S1C | 92.19(5) |
| N11–Co1–N11A | 180.00 | N11A–Co1–S1C | 87.81(5) |
| N1A–Co1–S1B | 91.64(5) | S1B–Co1–S1C | 180.00 |
| N1–Co1–S1B | 88.36(5) | | |

Table S3. Shortest intrachain and interchain distances in **1** and **2**.

| | Intrachain distance | Interchain distance |
|---|---|---|
| **1** $[Co(NCS)_2(N\text{-methylaniline})_2]_n$ | 5.6945 Å | 8.876 Å |
| **2** $[Co(NCS)_2(\text{aniline})_2]_n$ | 5.6539 Å | 6.9555 Å |

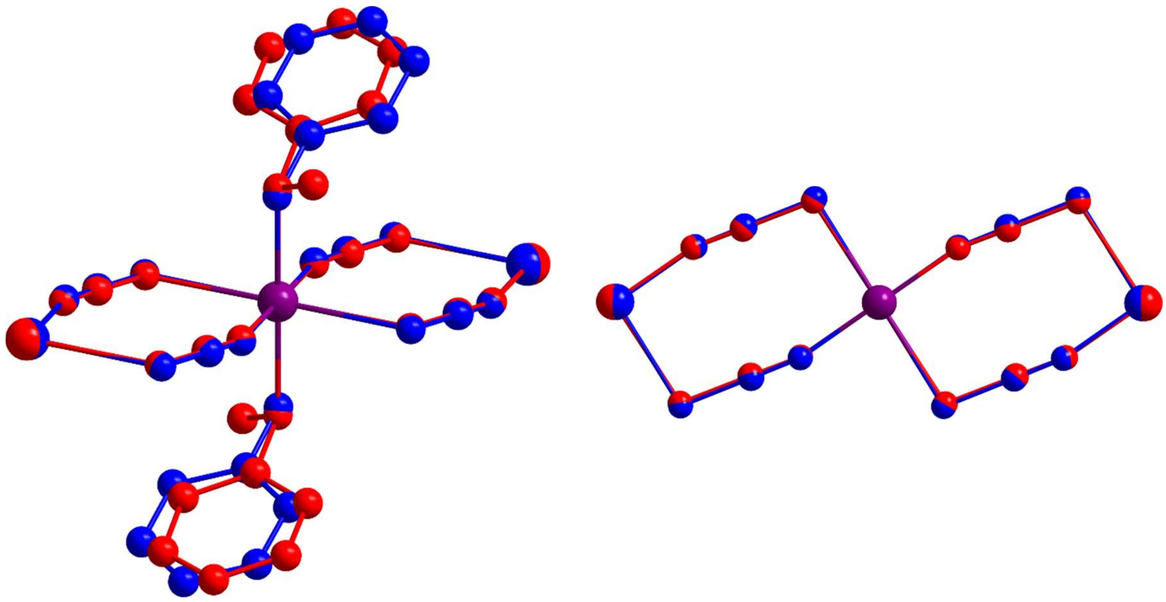

Fig. S2. Structural overlay of the octahedral Co(II) center (purple) in **1** (red) and **2** (blue) from two different perspectives (left: side view; right: top view; hydrogen atoms as well as the coligands in the top view have been omitted for clarity).

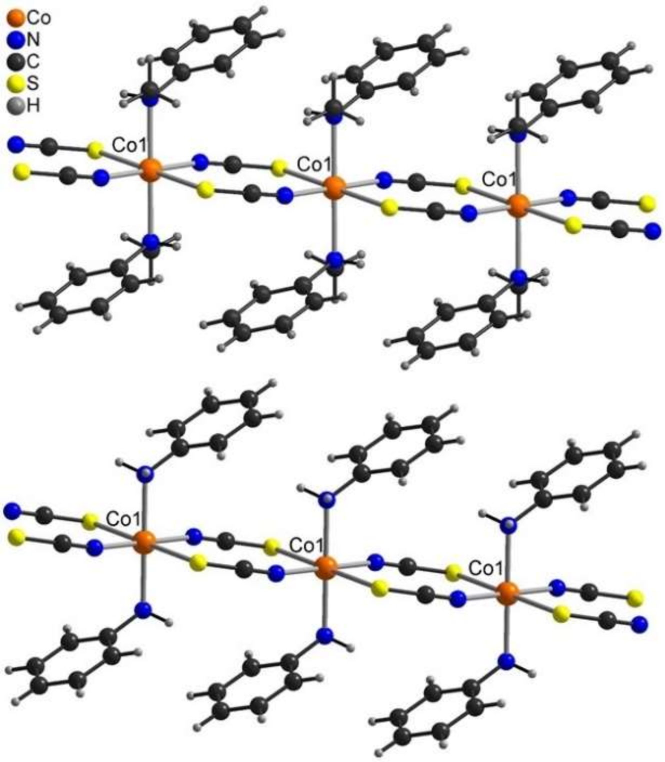


Fig. S3. View of a part of a chain in Co(NCS)$_2$(*N*-methylaniline)$_2$]$_n$ (**1**, top) and [Co(NCS)$_2$(aniline)$_2$]$_n$ (**2**, bottom).

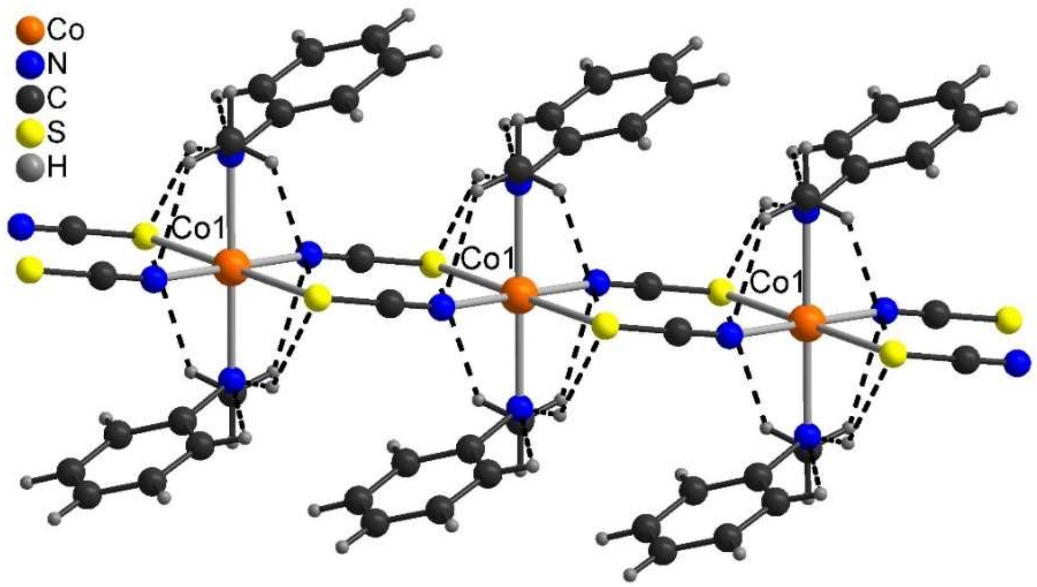


Fig. S4. View of a part of a chain of **1** with intrachain N–H···S, C–H···S, and C–H···N hydrogen bonding shown as dashed lines.

Table S4. Hydrogen bonds (Å and °) for **1**.

| D–H···A | *d*(D–H) | *d*(H···A) | *d*(D···A) | <(D–H···A) |
|---|---|---|---|---|
| N11–H11···S1B | 0.88 | 2.85 | 3.375(2) | 119.8 |
| C17–H17B···N1A | 0.98 | 2.61 | 3.140(3) | 114.0 |
| C17–H17C···S1C | 0.98 | 2.85 | 3.484(3) | 123.3 |

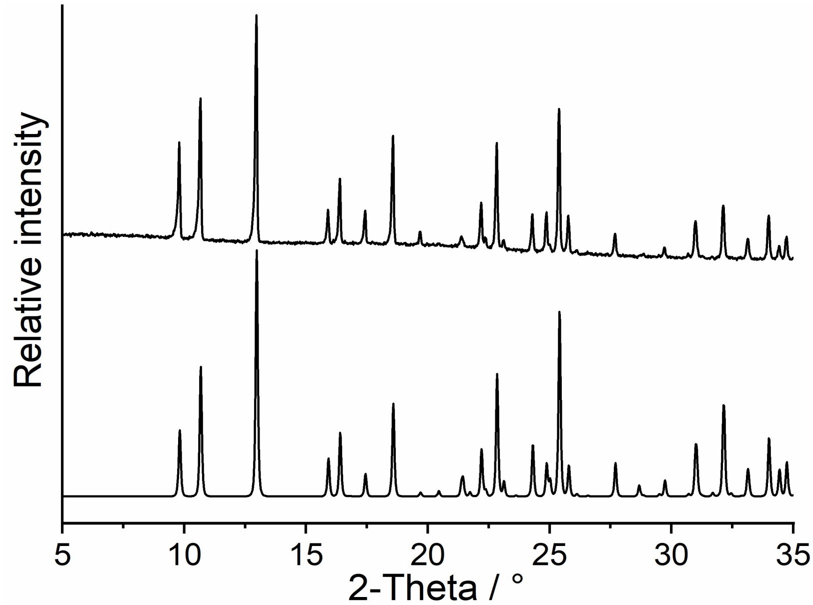


Fig. S5. Experimental (top) and calculated PXRD pattern (bottom) of **1**.

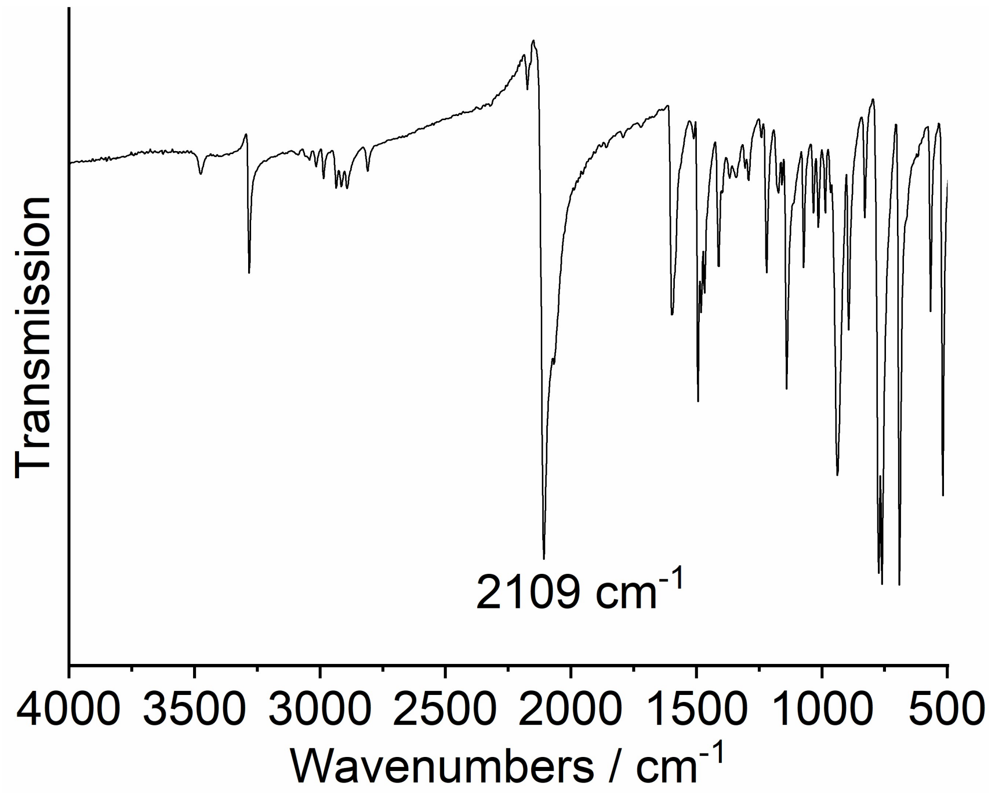


Fig. S6. IR spectrum of **1**. Given is the value of the CN stretching vibration.

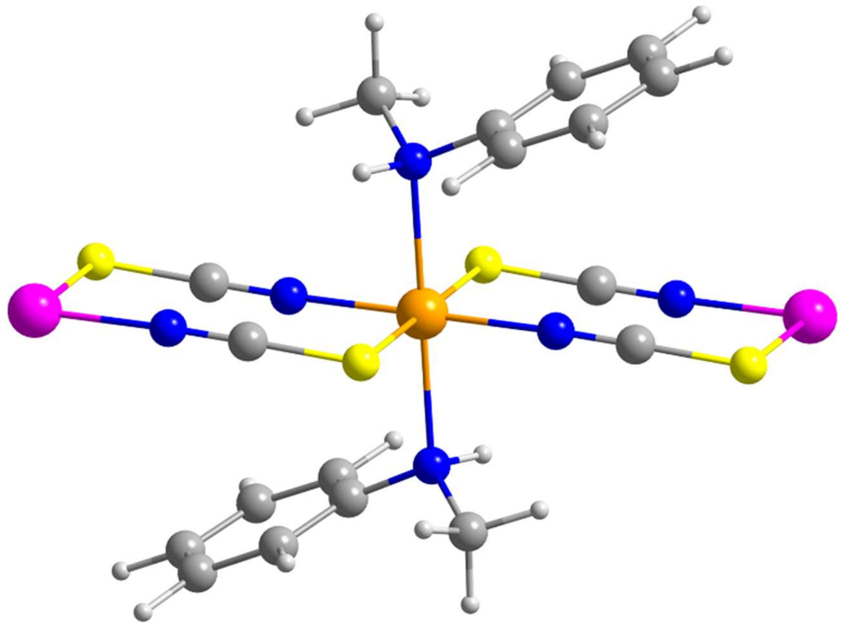

**Co1**

Fig. S7. Mononuclear model structure for *ab initio* calculations of **1** (Co – orange; Zn – pink).

Table S5. Relative CASSCF and CASPT2 energies (in cm$^{-1}$) of all quartet and the nine lowest doublet states for the mononuclear Co(II) computational model **Co1** (see Fig. S7) for **1**.

| 2*S*+1 | Term | | **Co1** | |
|---|---|---|---|---|
| | | | CASSCF | CASPT2 |
| 4 | $^4F$ | $^4T_{1g}$ | 0 | 0 |
| | | | 132 | 112 |
| | | | 880 | 795 |
| | | $^4T_{2g}$ | 5436 | 6389 |
| | | | 6296 | 7239 |
| | | | 8568 | 9631 |
| | | $^4A_{2g}$ | 14420 | 16466 |
| | $^4P$ | $^4T_{1g}$ | 21182 | 18760 |
| | | | 21888 | 19270 |
| | | | 25036 | 22702 |
| 2 | $^2G$ | | 12445 | 9633 |
| | | | 15824 | 12824 |
| | | | 17424 | 15451 |
| | | | 19036 | 16321 |
| | | | 19333 | 16773 |
| | | | 19783 | 16903 |
| | | | 20576 | 17665 |
| | | | 20905 | 17878 |
| | | | 24085 | 20898 |
| | … | | ... | ... |

Table S6. Relative energies (in $cm^{-1}$) for the Kramers doublets of the $^{4}T_{1g}$ multiplet as obtained from *ab initio* CASSCF/CASPT2/RASSI-SO calculations for **Co1** in comparison with the corresponding values from literature for $[Co(NCS)_2(aniline)_2]_n$ (denoted **Co2**).

| Kramers doublet | **Co1** | **Co2** |
|---|---|---|
| 1 | 0 | 0 |
| 2 | 230 | 228 |
| 3 | 459 | 478 |
| 4 | 744 | 819 |
| 5 | 1314 | 1141 |
| 6 | 1412 | 1268 |

Table S7. Relative energies (in $cm^{-1}$) for the first two Kramers doublets (KDs) of the $^{4}T_{1g}$ multiplet as obtained from *ab initio* CASSCF/CASPT2/RASSI-SO calculations for **Co1** together with the corresponding Cartesian components of the *g* tensor ($S_{eff}$ = 1/2)

| | KD1 | KD2 |
|---|---|---|
| $E$ ($cm^{-1}$) | 0 | 230 |
| $g_x$ | 2.303 | 0.643 |
| $g_y$ | 3.848 | 1.589 |
| $g_z$ | 6.600 | 4.225 |

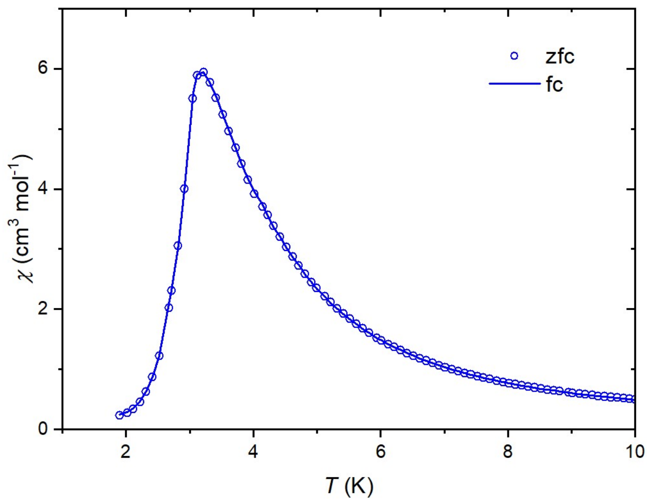


Fig. S8. Zero-field cooled (circles) and field cooled (line) susceptibility for **1** measured at 30 Oe.

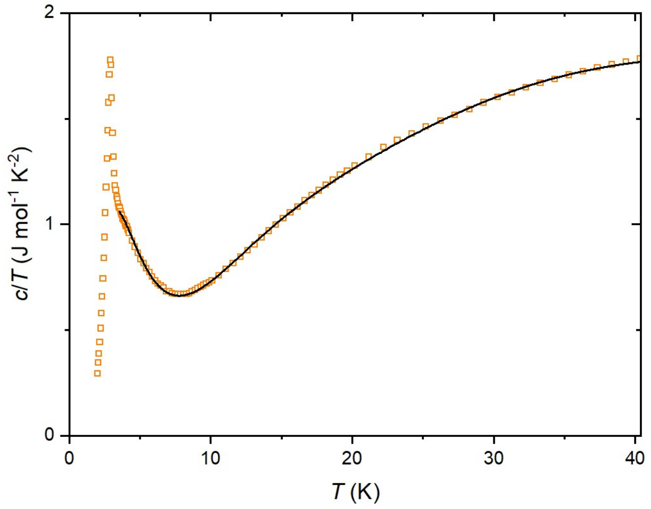


Fig. S9. Specific heat for **1** in the temperature range of 2-40 K. The solid line represents a fit (see main text and ref. 79).

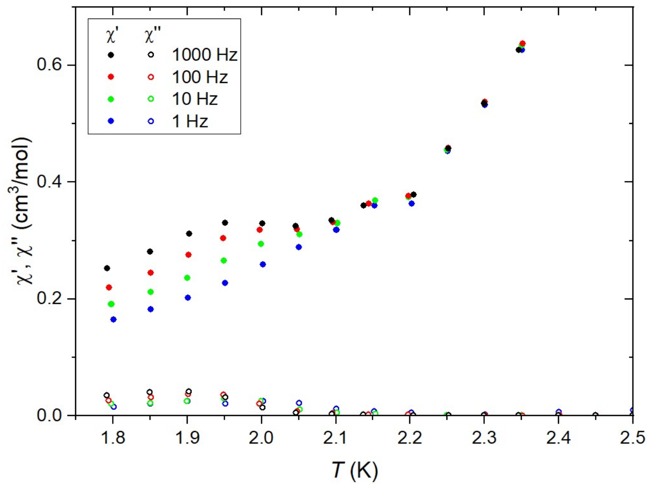


Fig. S10. Temperature dependence of the in-phase (•) and out-of-phase (◦) ac susceptibility for **1** at zero dc field.

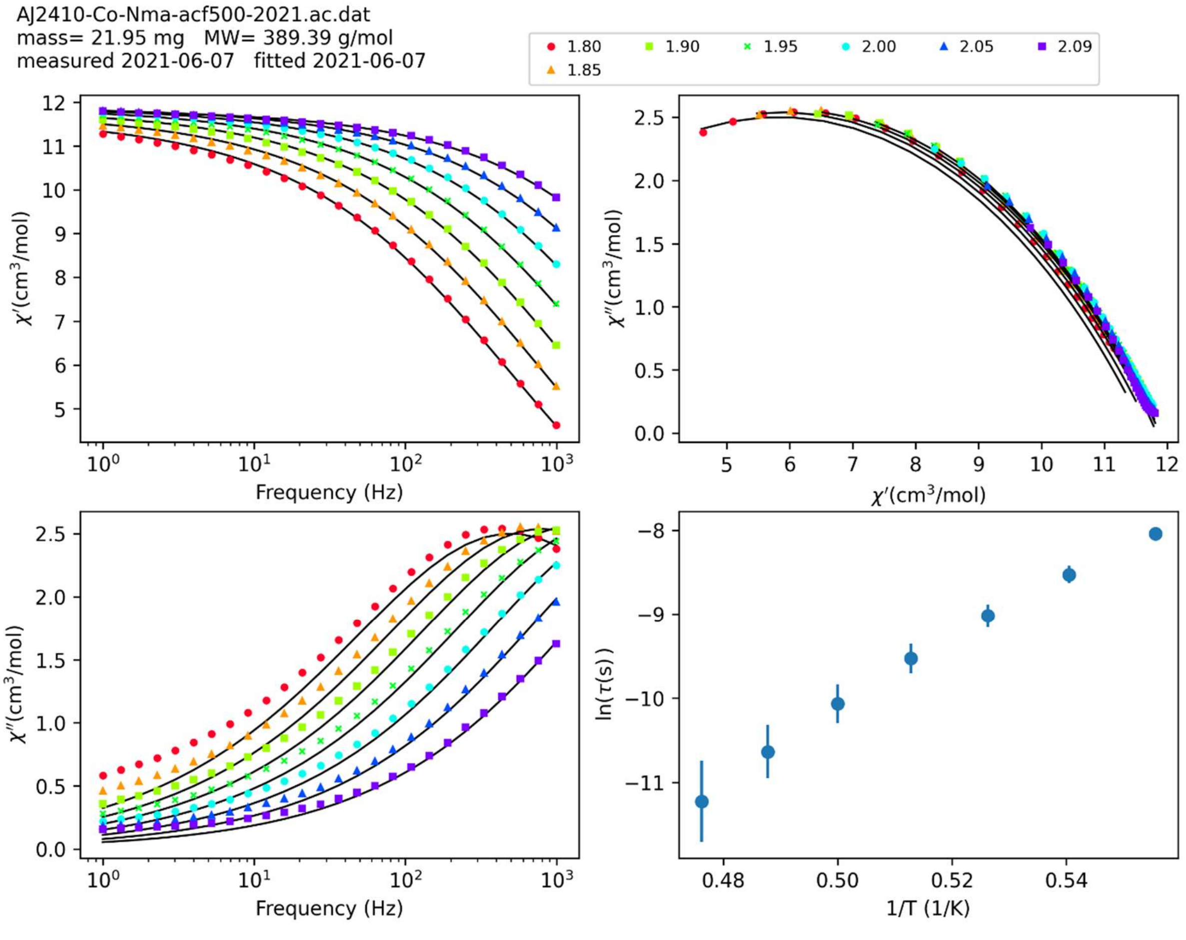


Fig. S11. Ac susceptibility measured for **1** at a dc field of 500 Oe and its analysis using a single-mode Cole–Cole model.

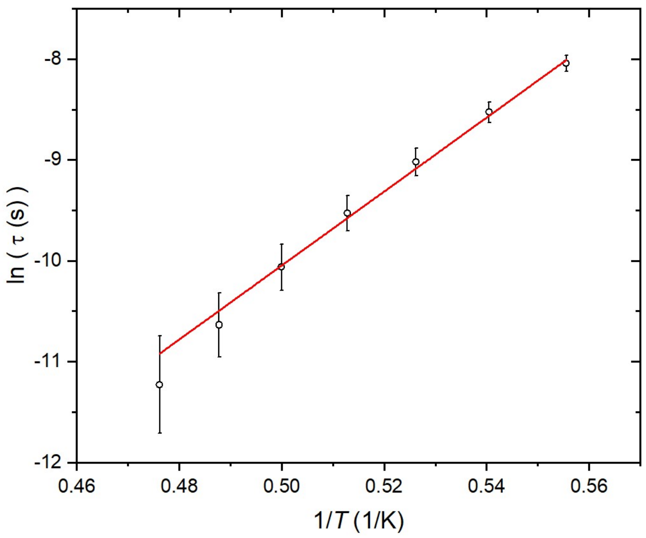


Fig. S12. Magnetic relaxation times measured for **1** at $H_{dc}$ = 500 Oe (circles) with a line fitted according to the Arrhenius equation (red line).

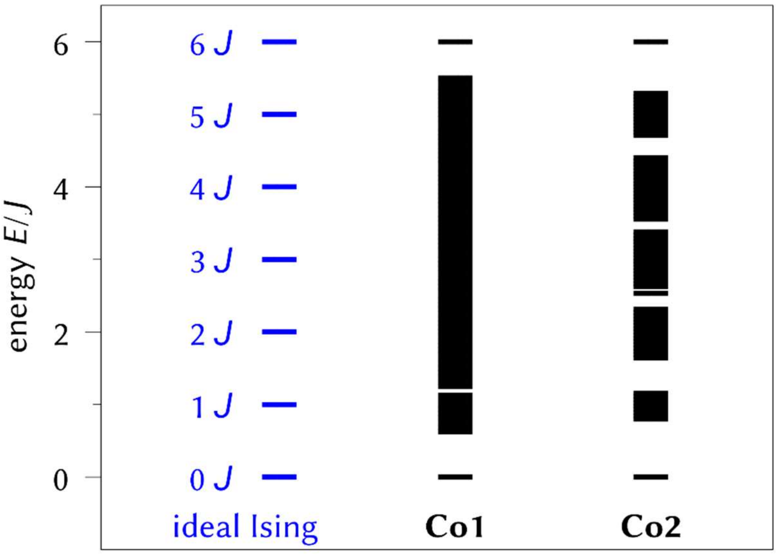


Fig. S13. Depiction of spin states as obtained by POLY_ANISO simulations employing the *ab initio* single-ion calculations of **Co1** and **Co2**, representing the compounds **1** and **2**, respectively. The spin states marked in blue describe the expected multiplet splitting for an ideal Ising anisotropy. For these simulations, a 12-membered spin ring coupling scheme was used, and the resulting spin state energies have been scaled to 6$J$, where $J$ represents the Ising coupling constant ($S_{eff}$ = 1/2).

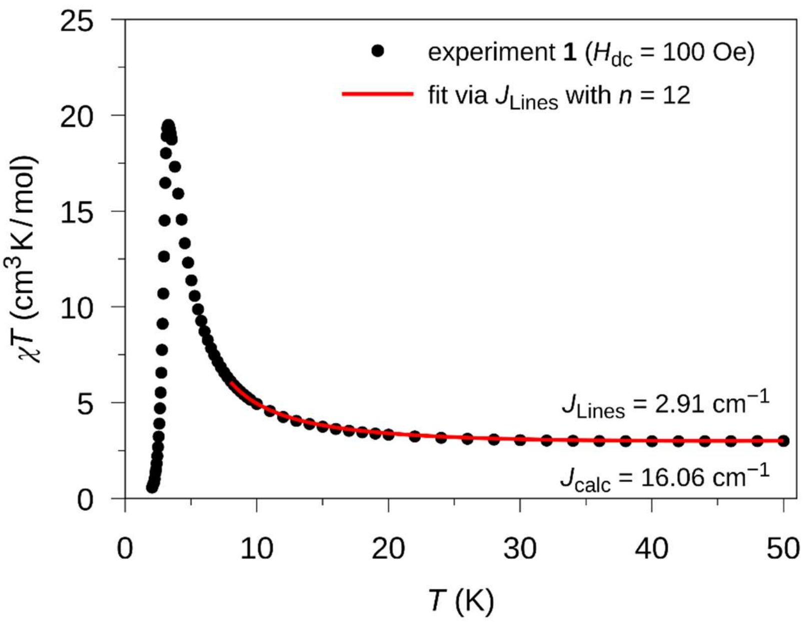


Fig. S14. Temperature dependence of the experimental magnetic susceptibility for **1** under an applied dc field of 100 Oe (•). The red line represents the simulation of a 12-membered spin ring that gives the best fit to the experimental data ($J_{Lines}$ = 2.91 cm$^{-1}$) in the temperature range of 8-50 K, based on the *ab initio* results obtained for the mononuclear model structure **Co1** and the Lines model as it is implemented in the POLY_ANISO program.

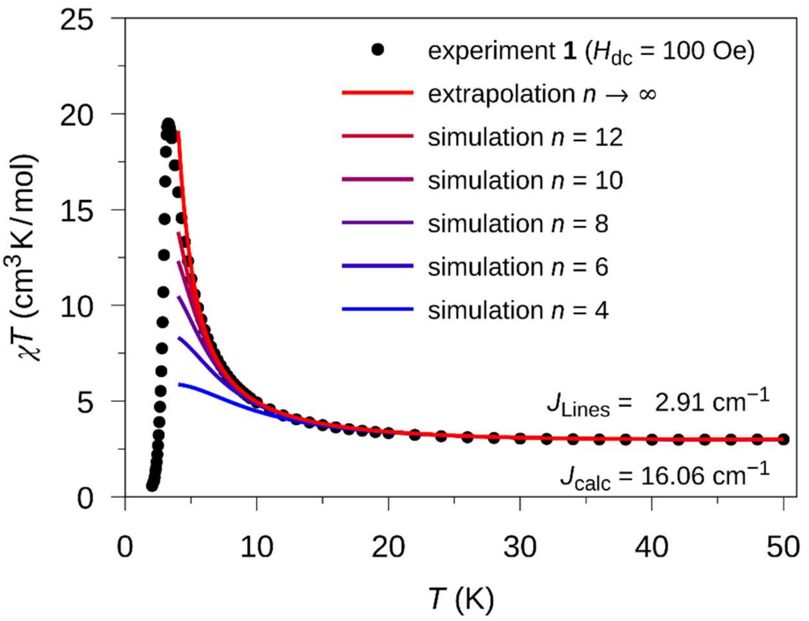


Fig. S15. Temperature dependence of the experimental magnetic susceptibility for **1** under an applied dc field of 100 Oe (•). Colored lines represent the simulation of $n$-membered spin rings without an interchain interaction ($zJ$ = 0) in dependence of $n$ based on the *ab initio* results obtained for the mononuclear model structure **Co1** and the Lines model as it is implemented in the POLY_ANISO program. The red line shows the extrapolation for $n \to \infty$.

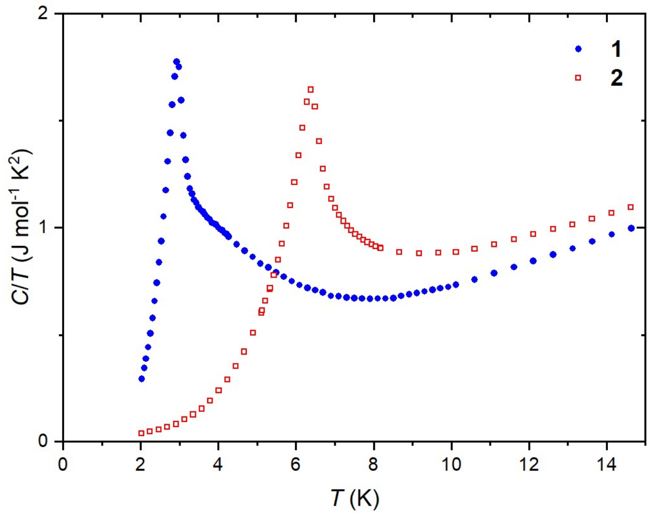


Fig. S16. Comparison of specific heat for $[Co(NCS)_2(\textit{N}\text{-methylaniline})_2]_n$ (**1**) and $[Co(NCS)_2(\text{aniline})_2]_n$ (**2**).

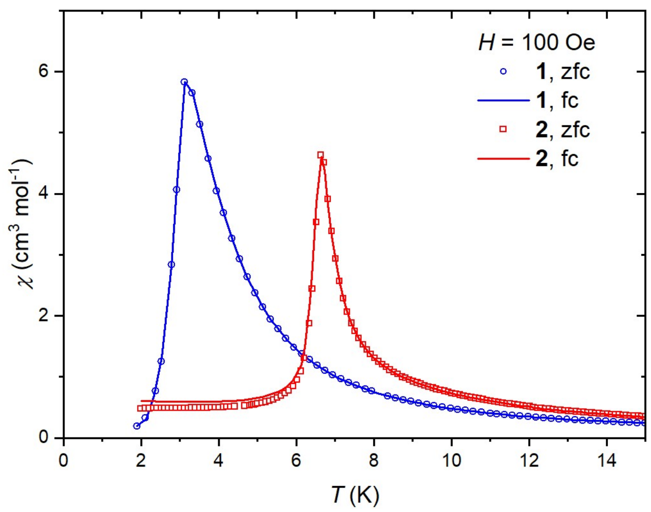


Fig. S17. Comparison of low-field susceptibility for **1** and **2**.

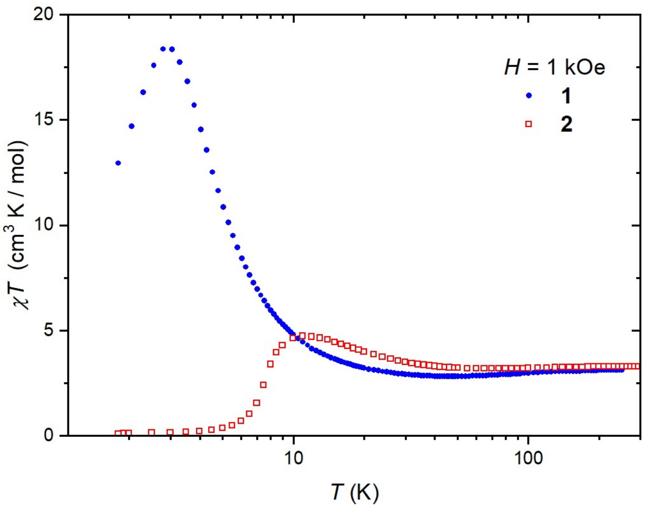


Fig. S18. Comparison of the susceptibility temperature product for **1** and **2**.

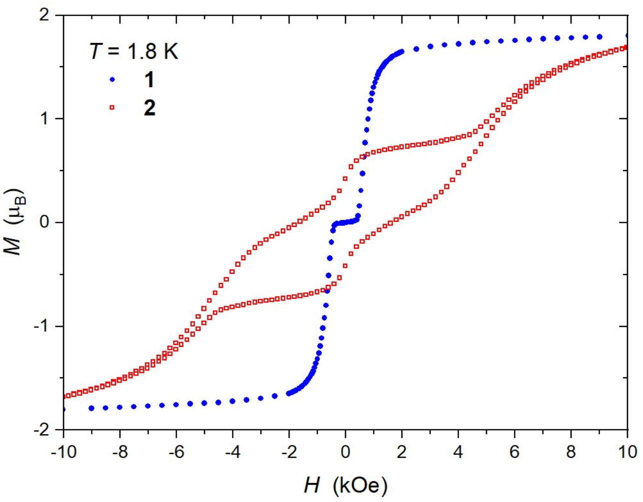


Fig. S19. Comparison of the field dependence of the magnetization at 1.8 K for **1** and **2**.

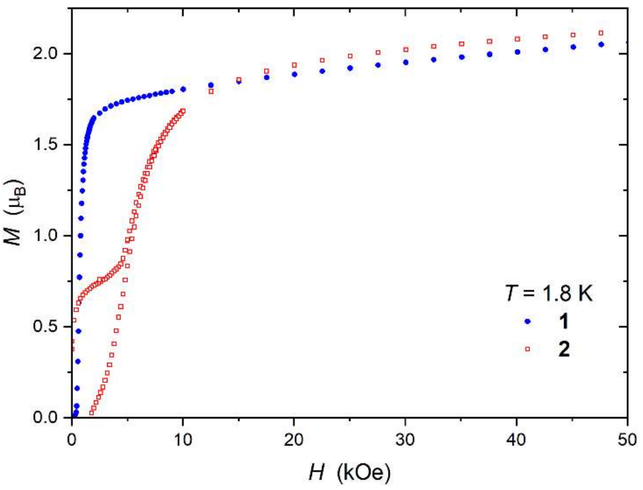

Fig. S20. Comparison of high-field magnetization at 1.8 K for **1** and **2**.
